# Identification and Analysis of Red Sea Mangrove (*Avicennia marina*) microRNAs by High-Throughput Sequencing and Their Association with Stress Responses

**DOI:** 10.1371/journal.pone.0060774

**Published:** 2013-04-08

**Authors:** Basel Khraiwesh, Ganesan Pugalenthi, Nina V. Fedoroff

**Affiliations:** 1 Center for Desert Agriculture, Division of Biological and Environmental Sciences and Engineering, King Abdullah University of Science and Technology, Thuwal, Kingdom of Saudi Arabia; 2 Bioscience Core Laboratory, King Abdullah University of Science and Technology, Thuwal, Kingdom of Saudi Arabia; 3 Evan Pugh Professor, Huck Institutes of the Life Sciences, Penn State University, University Park, Pennsylvania, United States of America; TGen, United States of America

## Abstract

Although RNA silencing has been studied primarily in model plants, advances in high-throughput sequencing technologies have enabled profiling of the small RNA components of many more plant species, providing insights into the ubiquity and conservatism of some miRNA-based regulatory mechanisms. Small RNAs of 20 to 24 nucleotides (nt) are important regulators of gene transcript levels by either transcriptional or by posttranscriptional gene silencing, contributing to genome maintenance and controlling a variety of developmental and physiological processes. Here, we used deep sequencing and molecular methods to create an inventory of the small RNAs in the mangrove species, *Avicennia marina*. We identified 26 novel mangrove miRNAs and 193 conserved miRNAs belonging to 36 families. We determined that 2 of the novel miRNAs were produced from known miRNA precursors and 4 were likely to be species-specific by the criterion that we found no homologs in other plant species. We used qRT-PCR to analyze the expression of miRNAs and their target genes in different tissue sets and some demonstrated tissue-specific expression. Furthermore, we predicted potential targets of these putative miRNAs based on a sequence homology and experimentally validated through endonucleolytic cleavage assays. Our results suggested that expression profiles of miRNAs and their predicted targets could be useful in exploring the significance of the conservation patterns of plants, particularly in response to abiotic stress. Because of their well-developed abilities in this regard, mangroves and other extremophiles are excellent models for such exploration.

## Introduction

Small 20–24 nucleotides (nt) non-coding RNAs are important regulators of gene expression, causing either transcriptional gene silencing (TGS) or posttranscriptional gene silencing (PTGS) [1]–[3]. They were first described in *Caenorhabditis elegans* [4] and were subsequently found to be responsible for RNA interference (RNAi), co-suppression, gene silencing, quelling and plant PTGS [5]–[9]. Small RNAs regulate a variety of biological processes in plants by interfering with mRNA translation, directing mRNA cleavage or promoting the formation of compact, transcriptionally inactive chromatin. Several distinct size classes of small RNAs with dedicated functions have been identified, including microRNAs (miRNAs), small interfering RNAs (siRNAs), repeat-associated small interfering RNAs (ra-siRNAs), natural antisense transcript-derived small interfering RNAs (nat-siRNAs), trans-acting small interfering RNAs (ta-siRNAs), heterochromatic small interfering RNAs (hc-siRNAs), secondary transitive small interfering RNAs, primary small interfering RNAs, and long small interfering RNAs (lsiRNAs) [10]–[12].

Small RNAs with specific sizes and functions have been identified by high-throughput sequencing in diverse plant species, including *Arabidopsis*, rice, barley, peanuts, grapevine, olive, *Medicago* and other plants [13]–[26]. Comparative studies on small RNAs across species have revealed the existence of highly conserved miRNAs and siRNAs with important regulatory functions in both plants and animals [27], [28]. Small RNAs are derived from partially double-stranded RNA (dsRNA) precursors by the action of ribonuclease III-family enzymes designated Dicers and Dicer-like (DCL) proteins [3], [29]. The small RNA duplexes generated by Dicer activity have a characteristic 2-nucleotide overhang at the 3′ end due to offset cleavage of the complementary strands by Dicers and DCLs. In plants these 3′ overhangs are stabilised by 2′-O-methylation [30]–[33]. One strand of the processed small RNA duplex subsequently associates with an Argonaute family protein and is incorporated into an RNA-induced silencing complex (RISC) that scans for nucleic acids complementary to the loaded small RNA to execute its function [34]–[37]. In plants, small RNAs act to silence genes by mediating RNA cleavage [38]–[41], translational repression [42]–[44], histone modification and DNA methylation [2], [45], [46]. RNA slicing and translational repression control gene expression post-transcriptionally, whereas histone modification and DNA methylation inhibit gene expression at the transcriptional level.

Dominating tropical intertidal zones and estuaries and evolutionarily adapted to tolerate flooding, anoxia, high temperatures, wind, and high and extremely variable salt conditions in typically resource-poor environments, mangrove ecosystems are comprised of halophytes, predominantly trees [47]–[50]. There are about 20 million hectares of mangroves in Asia, Oceania, Africa, the Americas and the Middle East [51]–[53]. Mangroves play an important role in coastal protection, maintenance of water quality and biodiversity [50]. Generally, mangrove forests are heavily exploited due to excessive wood gathering, fishpond operations, mining, and development of coastal areas and disposal of pollutants [50]. Mangroves exhibit several physiological strategies for handling salt, ranging from salt excretion (e.g., *Avicennia*, *Aegicaras*, *Sonneratia*) to salt balance regulation (e.g., *Rhizophora*, *Bruguiera*, *Xylocarpus*) to hyperexclusion (e.g., *Heritiera*) [54].

Although miRNAs have been profiled in a wide variety of herbaceous plant species, they have been analyzed in only a few woody plant species, among them conifer, poplar, grapevine, citrus, and olive trees and peanut plants [13], [14], [18], [19], [21]–[23]. In particular, limited studies have been conducted on mangrove small RNAs [55], [56]. Here we used high-throughput Illumina Genome Analyzer IIx (GAIIx) technology to sequence small RNA transcriptomes of the mangrove species, *Avicennia marina*. We used qRT-PCR to validate and analyze the expression patterns of identified miRNAs and their target genes in different mangrove tissues. Based on sequence similarity or the secondary structure of precursors, we have identified 193 conserved_miRNAs and 26 novel miRNAs in the small RNA transcriptome of *Avicennia marina*. All targets regulated by *Avicennia marina* annotated miRNAs identified in this study were subjected to gene ontology (GO) analysis to identify gene functions.

## Materials and Methods

### Ethics Statement

No specific permits were required for the described field study. No specific permissions were required for this location and activities. The location is not privately-owned or protected in anyway and the field studies did not involve endangered or protected species.

### Plant Materials and RNA Isolation

Mangrove (*Avicennia marina*) samples were collected from the Red Sea Coast, Kingdom of Saudi Arabia. Tissue samples were immediately frozen in liquid nitrogen and stored at −80°C. Total RNA was extracted from tissue samples.

### Small RNA Library Construction and Sequencing

Total RNA was extracted from several mangrove tissues (roots, stems, leaves, buds, flowers, and seeds) using TRIzol reagent (Invitrogen, USA) following the manufacturer’s instructions. Total RNA from the different tissue samples was mixed in an equal fraction ratio. The mangrove small RNA library was prepared with TruSeq Small RNA Sample Prep Kit (Illumina, Inc.) using the illumina TruSeq small RNA sample preparation protocol according to the manufacturer’s instructions. Briefly, the RNA 3′ adapter was modified to target miRNAs and other small RNAs that had a 3′ hydroxyl group resulting from DCL cleavage. The adapters (3′ adapter: 5′-TGGAATTCTCGGGTGCCAAGG-3′ and 5′ adapter: 5′-GUUCAGAGUUCUACAGUCCGACGAUC-3′) were ligated to each end of the RNA molecule and a reverse-transcription (RT) reaction was carried out to generate single-stranded cDNA using RT primer (5′-GCCTTGGCACCCGAGAATTCCA-3′). The cDNA was then PCR amplified using the following primers: 5′-AATGATACGGCGACCACCGAGATCTACACGTTCAGAGTTCTACAGTCCGA-3′ and 5′-CAAGCAGAAGACGGCATACGAGATCGTGATGTGACTGGAGTTCCTTGGCACCCGAGAATTCCA-3′. Following PCR amplification, the small RNA was purified from the gel and concentrated by ethanol precipitation. Next, the amplified cDNA construct was subjected to 6% (w/v) polyacrylamide gel electrophoresis (PAGE). The small RNA band containing the 30 nt RNA fragment with both adapters (157 nt) was purified by size fractionation followed by gel elution and precipitation. Finally, the small RNA library was sequenced directly using the Illumina GAIIx technology (Illumina, Inc.).

### Small RNA Analysis: Identification of miRNAs and miRNA Target Prediction

The raw sequences were filtered as follows: i) poor quality reads, which included reads with “N” characters and reads of low sequence complexity (reads with fewer than 3 different bases), were removed using an in-house script written in Perl; ii) reads without reliable 3' adapters were discarded from the dataset; iii) 3' adapters were removed using an in-house script written in Perl; iv) reads outside the 18–25 nt size range were removed; and v) BLAST searches were performed against *Rfam* database (Rfam 11.0) [57] and plant repeat databases to discard abundant non-coding RNAs (rRNA, tRNA, snRNA, and snoRNA) or mRNAs degradation products (http://rfam.sanger.ac.uk/and http://plantrepeats.plantbiology.msu.edu/).

We performed a BLASTN search on each unique sequence remaining after the filtering steps against known mature and precursor miRNAs (pre-miRNAs) from other plant species deposited in the miRBase database (http://www.mirbase.org/) [58]. Only perfectly matched sequences were considered to be conserved miRNAs. Conserved miRNAs having perfect matches to mangrove cDNA sequences (deposited in the Mangrove Transcriptome Database (MTDB), http://mangrove.illinois.edu, a web-based platform providing transcriptome information from 28 mangrove species [55]) were subjected to stem-loop structure prediction using *Mfold* web server (http://mfold.rna.albany.edu/?q=mfold) [59]. Predictions were made using RNA sequences containing 50–200 nucleotides on either side of the candidate miRNA. In case no apparent local foldback structure was predicted for a given sequence, larger upstream and downstream sequences were submitted for *Mfold* analysis. The criteria used to identify candidate structured precursors were those suggested by [60].

Target genes of the miRNAs were predicted using the online tool *psRNATarget*, a small RNA target analysis server for plants (http://plantgrn.noble.org/psRNATarget/) [61]. This tool uses an iterative parallel Smith-Waterman algorithm and a weighted scoring scheme in which mismatched bases are penalized according to their type and location in the alignment. Mismatches to the 5′ and central regions of the miRNA were preferentially penalized compared with mismatches to the 3′ region of the miRNA. Functions were assigned to the predicted targets manually based on the function of the best hit from the BLAST homology search against TAIR10 database (http://arabidopsis.org/) and the MTDB database [55].

The raw and processed sequencing data have been deposited into NCBI Gene Expression Omnibus (GEO) under accession number GSE43669.

### Reverse Transcription (RT)-PCR and Real Time PCR of Mangrove miRNAs and their Target Genes

We used a published method for qRT-PCR of small RNAs [62] with some modifications. Briefly, for each miRNA, a specific stem-loop RT primer was used for reverse transcribing from purified total RNA of mangrove tissues (roots, stems, leaves, flowers, and seeds) for cDNA synthesis. The cDNA was then used for real time PCR on a LightCycler 480 instrument system (Roche, Germany) using SYBR green-based real time PCR with miRNA-specific forward primer and universal reverse primer (Table S1). U6 was used as the internal control. For real time PCRs of conserved and novel miRNA target genes, total RNA samples from the same mangrove tissues (roots, stems, leaves, flowers, and seeds) were treated with DNase I (Fermentas, USA) and reverse-transcribed into first-strand cDNA using the Superscript III First-Strand Synthesis System (Invitrogen, USA). Real time PCR reactions were performed on LightCycler 480 instrument system (Roche, Germany) using SYBR green-based real time PCR with gene-specific primers according to the manufacturer’s instructions. Constitutively expressed *18S rRNA* gene (accession no. Y17766) was used as reference gene for normalization. All reactions were run in triplicate. The primers for subsequent real time PCR reactions are reported in Table S1.

### Detection of miRNA Target Cleavage Products

Synthesis of 5′ RACE-ready cDNAs was carried out using the BD Smart RACE cDNA amplification kit (CLONTECH, USA). Subsequent PCR reactions were performed using the UPM Primer-Mix supplied with the kit in combination with gene-specific primers derived from miRNA target genes (Table S1). Amplification products corresponding to the size of the expected cleavage products were then gel-purified, cloned and sequenced.

### Gene Ontology Analysis

To identify miRNA target functions and classifications, as well as the metabolic regulatory networks associated with mangrove miRNAs and their targets, we conducted GO analysis by running a BLASTX search for each target sequence against UniProt database [63]. The best hits were used to validate the target gene functions and metabolic pathways regulated by miRNAs. The molecular functions of the gene products and the subcellular locations where these products are located were obtained from UniProt-GO Annotation database [64].

## Results and Discussion

### Construction and Sequence Analysis of a Small RNA Library from *Avicennia marina*

**Figure 1:**
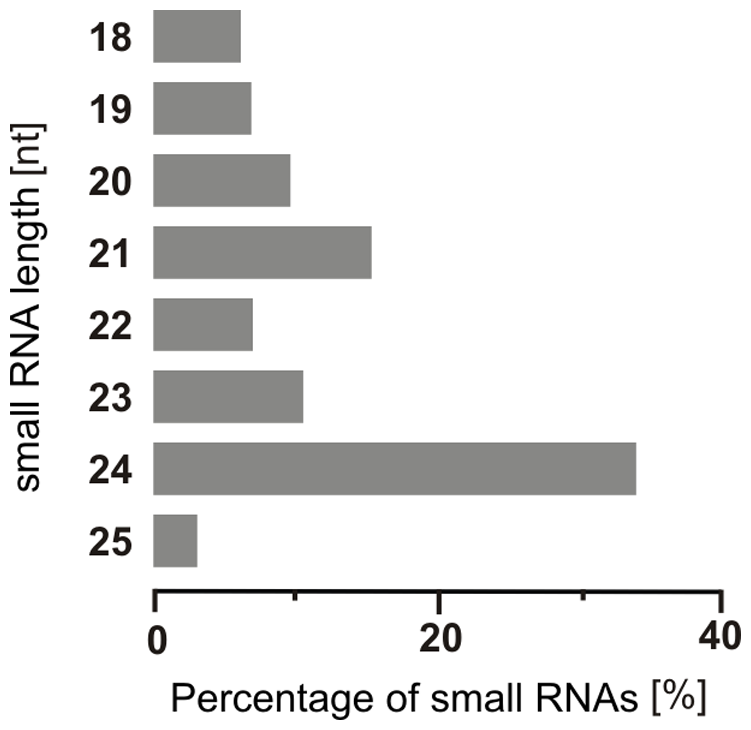
Length distribution and abundance of sequenced small RNAs from *Avicennia marina*.

High-throughput sequencing offers a powerful means for quantitative and qualitative profiling of small RNA populations and it is convenient for exploring small RNAs in plant species such as *Avicennia marina* for which limited genome information is available. In this study, a small RNA cDNA library was generated using a pooled sample containing roots, stems, leaves, buds, flowers, and seeds. The library was designed to include RNAs with the size and the biochemical signatures (5′ phosphate and 3′ hydroxyl groups) of DCL cleavage products. We obtained a total of 36,503,464 unfiltered reads. After removing the adaptor/acceptor sequences, we filtered the reads to remove species outside the 18–25 nt size range (the typical size range for DCL-derived products), species with low sequence complexity (less than 3 different bases), and species with non-coding RNA matches. That left 1,978,971 clean reads. Among the total reads, 1,837,251 were found to be similar to existing miRNAs (Table 1). The majority of the mangrove silencing small RNAs obtained in this study were 24 nt in size, accounting for 35% of silencing small RNAs, followed by 21 nt (16%), 23 nt (12%), 20 nt (10%), and 22 nt (8%) (Figure 1). This result was consistent with those reported for other plant species, including *A. thaliana*, *M. truncatula*, *O. sativa*, *P. trichocarpa*, *A. hypogaea*, and *C. trifoliate* [13], [15], [18], [21], [23], [25].

**Table 1:** Summary statistics of small RNA sequencing in *Avicennia marina*.

	Sequences generated	Unique sequences
Raw reads	36,503,464	
Low quality reads removed	35,325,851	
Adaptors removed	16,606,288	3,779,879
Sequence outside 18–25 nt length removed	10,140,716	2,711,570
Sequences matching MTDB*	2,609,692	75,874
Non-coding RNA exact matches removed	1,978,971	32,198
Match known miRNAs**	1,837,251	193

* Mangrove Transcriptome Database.

** All possible miRNA sequence polymorphisms were counted.

Molecules of 24 nt in length that are processed by DCL3 are often the most abundant endogenous small plant RNAs, and the high percentage of 24 nt small RNAs may reflect the complexity of the mangrove genome, as 24 nt siRNAs are known to be involved in heterochromatin modification [3]; this may vary according to species, however. For example, 24 nt small RNAs are more abundant in *Arabidopsis*, rice, peanut, olive, and tomato [13]–[15], [24], whereas 21 nt small RNAs are more abundant in grapevine, wheat, conifers, and *Physcomitrella* [19], [65].

### Identification of Conserved miRNAs in *Avicennia marina*

Over the past several years, miRNAs have been studied extensively, with 25,141 miRNAs from 193 species deposited in the miRBase database, including 5,940 miRNAs from 67 plant species belonging to 1,558 different families (Release 19.0, August 2012) [58]. After removing repeat sequences, we used 32,198 unique small RNA sequences as queries in the miRBase database to search for potential miRNAs in *Avicennia marina*. On the basis of sequence similarity, we identified 193 conserved miRNAs belonging to 36 miRNA families (Table S2). As expected, most of the miRNAs we identified were highly conserved in diverse plant species (Table 2), suggesting that the ancient regulatory pathways mediated by evolutionarily conserved miRNAs are present in mangrove species. Most of the miRNA families conserved between *Arabidopsis*, *Populus* and rice were also identified in our dataset (Table 2; Table S2). Sequence analysis indicated that conserved miRNAs in *Avicennia marina* had higher similarity to their homologs in *Populus* than in *Arabidopsis* and rice. Lee et al. [56] used a bioinformatic approach for miRNA prediction from ESTs for the mangrove *Bruguiera* spp. Candidate miRNA precursors, which potentially belong to the miR156/157, miR396 and miR529 families were identified [56]. Dassanayake et al. [55] presented 12 miRNA families using MTDB annotations coupled with the BLAST tool incorporated in MTDB. Because MTDB largely originated from the analysis of a size-selected poly-A enriched cDNA library designed to identify protein-coding genes, the frequency with which mature miRNAs are expected to be present in MTDB is low [55].

**Table 2:** Highly conserved miRNAs in *Avicennia marina*.

MiRNAfamily	Sequence (5′-3′)	Length(nt)	Conserved in other plants											
			ath	ptr		bdi		gma		zm	osa		vvi	
miR156	UGACAGAAGAGAGGGAGCAC	20	−	++++	−		−		−			−		++++
	UGACAGAAGAGAGUGAGCAC	20	++++	++++	++++		++++		++++			++++		++++
	UUGACAGAAGAUAGAGAGCAC	21	−	++++	−		++++		−			−		++++
miR159	UUUGGAUUGAAGGGAGCUCUA	21	++++	++++	−		++++		−			−		++++
	UUUGGAUUGAAGGGAGCUCUG	21	−	−	−		−		++++			++++		−
miR160	UGCCUGGCUCCCUGUAUGCCA	21	++++	++++			++++		++++			++++		++++
	UGCCUGGCUCCCUGUAUGCCG	21	−	−	−		−		++++			++++		−
miR164	UGGAGAAGCAGGGCACGUGCA	21	++++	++++	++++		++++		++++			++++		++++
miR166	GGAAUGUUGGCUGGCUCGAGG	21	−	−	−		−		++++			++++		−
	GGAAUGUUGUCUGGCUCGAGG	21	−	−	−		++++		++++			++++		−
	UCGGACCAGGCUUCAAUCCCU	21	−	−	−		−		++++			++++		−
	UCGGACCAGGCUUCAUUCCC	20	−	−	−		++++		++++			−		−
	UCGGACCAGGCUUCAUUCCCC	21	++++	++++	++++		++++		++++			++++		++++
	UCGGACCAGGCUUCAUUCCUC	21	−	−	−		−		++++			++++		−
	UCUCGGACCAGGCUUCAUUCC	21	−	−	++++		++++		−			−		−
miR167	UGAAGCUGCCAGCAUGAUCUA	21	++++	++++	++++		++++		++++			++++		++++
	UGAAGCUGCCAGCAUGAUCUG	21	−	++++	−		++++		++++			++++		++++
	UGAAGCUGCCAGCAUGAUCUGA	22	−	−	++++		++++		−			−		−
	UGAAGCUGCCAGCAUGAUCUU	21	−	++++	−		++++		−			−		−
miR168	UCGCUUGGUGCAGAUCGGGAC	21	−	−	++++		−		++++			++++		−
	UCGCUUGGUGCAGGUCGGGAA	21	++++	++++	−		++++		−			−		++++
miR169	CAGCCAAGGAUGACUUGCCGG	21	++++	++++	++++		++++		++++			++++		++++
	UAGCCAAGGAUGACUUGCCUA	21	−	++++	++++		−		++++			++++		++++
miR171	UGAUUGAGCCGUGCCAAUAUC	21	−	++++	++++		++++		++++			++++		++++
	UGUUGGCUCGGCUCACUCAGA	21	−	−	−		−		++++			++++		−
	UUGAGCCGCGCCAAUAUCACU	21	−	−	−		++++		−			−		++++
	UUGAGCCGUGCCAAUAUCACG	21	++++	++++	−		++++		−			−		−
miR172	AGAAUCUUGAUGAUGCUGCA	20	−	−	−		++++		++++			−		−
	AGAAUCUUGAUGAUGCUGCAU	21	++++	++++	++++		++++		−			++++		−
miR319	UUGGACUGAAGGGAGCUCCC	20	−	++++	−		++++		−			−		−
	UUGGACUGAAGGGAGCUCCCU	21	++++	−	−		++++		−			−		++++
	UUGGACUGAAGGGUGCUCCC	20	−	−	−		−		++++			++++		−
miR390	AAGCUCAGGAGGGAUAGCGCC	21	++++	++++	++++		++++		++++			++++		++++
miR393	UCCAAAGGGAUCGCAUUGAUC	21	−	++++	++++		++++		−			++++		++++
	UCCAAAGGGAUCGCAUUGAUCC	22	++++	−	−		++++		++++			−		−
	UCCAAAGGGAUCGCAUUGAUCU	22	−	−	−		−		++++			++++		−
miR394	UUGGCAUUCUGUCCACCUCC	20	++++	++++	++++		++++		++++			++++		++++
miR396	GGUCAAGAAAGCUGUGGGAAG	21	−	−	−		−		++++			++++		−
	UUCCACAGCUUUCUUGAACUG	21	++++	++++	++++		++++		++++			++++		++++
	UUCCACAGCUUUCUUGAACUU	21	++++	++++	++++		++++		++++			++++		−
miR397	UCAUUGAGUGCAGCGUUGAUG	21	++++	++++	++++		++++		−			++++		++++
miR398	UGUGUUCUCAGGUCACCCCUU	21	++++	++++	−		++++		−			++++		++++
	UGUGUUCUCAGGUCGCCCCUG	21	−	++++	++++		++++		−			++++		++++
miR399	UGCCAAAGGAGAAUUGCCCUG	21	−	++++	++++		−		++++			++++		++++
	UGCCAAAGGAGAGUUGCCCUA	21	−	++++	−		−		−			++++		−
	UGCCAAAGGAGAGUUGCCCUG	21	++++	−	−		++++		++++			++++		++++
	UGCCAAAGGAGAUUUGCCCGG	21	++++	++++	−		−		−			−		++++
miR403	UUAGAUUCACGCACAAACUCG	21	++++	++++	−		−		−			−		++++
miR408	AUGCACUGCCUCUUCCCUGGC	21	++++	++++	−		++++		−			−		++++
	CUGCACUGCCUCUUCCCUGGC	21	−	−	−		−		++++			++++		−
miR828	UCUUGCUCAAAUGAGUAUUCCA	22	−	++++	−		++++		−			−		++++
miR2111	UAAUCUGCAUCCUGAGGUUUA	21	++++	−	−		++++		−			−		−

The abbreviations represent the following: ath, *Arabidopsis thaliana*; ptr, *Populus trichocarpa*; bdi, *Brachypodium distachyon*; gma, *Glycine max;* zma, *Zea mays*; osa, *Oryza sativa*; vvi, *Vitis vinifera*. ++++, miRNA sequences of mangrove that were exactly identical to those of other species; -, miRNA sequences of mangrove that were not present in other species.

The secondary structures of conserved miRNAs were predicted using *Mfold* RNA folding platform. Due to the incompleteness of the mangrove genome, pre-miRNAs having characteristic secondary structures could only be predicted for miR156, miR159, miR171, miR396, mi397 and miR398 (Figure S1).

The relative frequencies of miRNAs in a library can be used as an index for estimating the relative abundance of miRNAs. Using high-throughput sequencing, we identified a large number of miRNA sequences, allowing us to determine the relative abundance of miRNAs in *Avicennia marina*. The frequencies of miRNA families varied from 1 (miR828) to 1,657,731 (miR166), indicating that expression varies significantly among different miRNA families. Counting redundant miRNA reads revealed that 11 of the 36 conserved miRNA families were represented by more than 1,000 reads in the mangrove dataset (Table S2). The miR166 (1,657,731 reads), miR319 (59,840 reads), miR396 (39,440 reads), miR159 (28,548 reads), miR156 (19,310 reads) and miR168 (16,725 reads) families were the most frequent in the library (Table S2). These results indicate that different members have clearly different expression levels in one miRNA family, probably because the expression is tissue- and/or developmental-stage specific. In comparison with other plant species, miR156, miR166 and miR168 in peanut, miR156a, miR168, miR528 and miR166a in *Brachypodium*, miR169b in wheat and miR169 in rice were the most frequently sequenced miRNAs, while miR156 in rice and wheat exhibited low abundance [66], [67]. In addition, miR156a was found to be highly expressed in *Medicago,* another legume species [25]. However, it is not known by the abundance of the same miRNAs differs markedly in different plant species.

### Prediction of Novel miRNAs in *Avicennia marina*

The most challenging problem in understanding plant miRNAs is identifying novel miRNAs. Three major approaches have been used for miRNA identification in plants: forward genetics, bioinformatic prediction, and direct cloning and sequencing. Only a few miRNAs were identified by forward genetic studies and predicting species-specific miRNAs using bioinformatics remains difficult. Thus, direct cloning and sequencing is the most effective method for plant miRNA identification.

To uncover additional mangrove-specific miRNA candidates within our sequenced set, we relied on MTDB, a platform providing transcriptome information from 28 mangrove species [55], because details of the mangrove genome sequence remain limited. In all, 26 small RNAs met our criteria as established according to Meyers et al. [60] and were considered to be putative novel mangrove miRNAs (Table 3). For eight of these candidate miRNAs, we found both miRNA and miRNA* sequences (Table 3; Figure S2). The detection of miRNAs* adding weight to the authenticity of the predicted miRNA candidates. These novel candidates displayed a concentrated length distribution between 18 to 22 nt. Precursors of these novel miRNAs had negative folding free energies ranging from −84 to −22 kcal mol^−1^, with an average of about −57 kcal mol^−1^ according to the *Mfold* RNA folding platform. These values are similar to the free energy values of other plant miRNA precursors (−59.5 kcal mol^−1^ in *A. thaliana* and −71.0 kcal mol^−1^ in *O. sativa*) and are much lower than the reported folding free energies of tRNAs or rRNAs [68].

**Table 3:** Novel miRNAs/miRNA*s predicted in *Avicennia marina*.

miRNA name	Sequence (5′-3′)	Length (nt)	Count	Precursor length (nt)	dG (kcal mol^−1^)
miR1	CUUCUAUAGUUUAGGUAACUU	21	1	55	−29.6
miR2.1	GUGAUGGGGAUAGAUCAUUGCA	22	188	118	−84
miR2.2	UUGUUGGUCUUCAACGAGGAAU	22	14	118	−84
miR2.2*	AUUCCUCGUUGAAGACCAACAA	22	4	118	−84
miR2.3	GUUGGUCUUCAACGAGGAAUU	21	16	118	−84
miR2.3*	AAUUCCUCGUUGAAGACCAAC	21	5	118	−84
miR2.4	UUGGUCUUCAACGAGGAAUUC	21	17	118	−84
miR2.4*	GAAUUCCUCGUUGAAGACCAA	21	8	118	−84
miR2.5	UGGUCUUCAACGAGGAAUUCCU	22	16	118	−84
miR3.1	AUGGGCAGGCAAGAGACAAC	20	2	146	−72
miR3.1*	GUUGUCUCUUGCCUGCCCAU	20	2	146	−72
miR3.2	UCCAUGGGCAGGCAAGAGA	19	2	146	−72
miR3.3	CUGAAUCCAUGGGCAGGCAAGA	22	2	146	−72
miR3.3*	UCUUGCCUGCCCAUGGAUUCAG	22	2	146	−72
miR3.4	GCUGCUGAAUCCAUGGGCAGGC	22	8	146	−72
miR3.4*	GCCUGCCCAUGGAUUCAGCAGC	22	8	146	−72
miR3.5	UGCUGCUGAAUCCAUGGGCAG	21	30	146	−72
miR4	UUAGAUUCACGCAUAAACUCG	21	1	119	−35.3
miR5	AGAAAAGAGAAAGAAAAGACA	21	1	173	−33.8
miR6	GAAUAGGAAGAUCUGAUGCCU	21	74	52	−22
miR7	CGGCAUCCGUCGAUUCAACG	20	4	72	−28.6
miR8	CGGGAAGAGUUAUCUUUUCU	20	119	64	−26.7
miR9	CUUUUAACGUUAUUUUAC	18	63	86	−27
miR10	GAUAGAGAGAGAGAGAGA	18	2	151	−70
miR11	GAUGAUGAUGAUGAUGAU	18	5	58	−20.4
miR12	UCGCGGCGACGUGGGCGG	18	272	46	−33.2
miR13.1	CAUCUCUCUCUCUCUCUCU	19	1	211	−58
miR13.2	CUCUCUCUCUCUCUCUCUCA	20	3	211	−58
miR14	UUGAUUAGAGUAGGGGUCGCG	21	16	96	−33.3
miR14*	AACCGCGACUCUUGUCAUAGU	21	5	96	−33.3
miR15	UUGAAUAUUAAGUAUGAA	18	1	244	−80
miR156.1	CUGACAGAAGAGAGUGAGCACA	22	3	88	−38
miR396.1	GAAGCUCAAGAAAGCUGUGGGA	22	1	95	−38
miR396.1*	UUCCACAGCUUUCUUGAACUUU	22	20	95	−38

**Figure 2:**
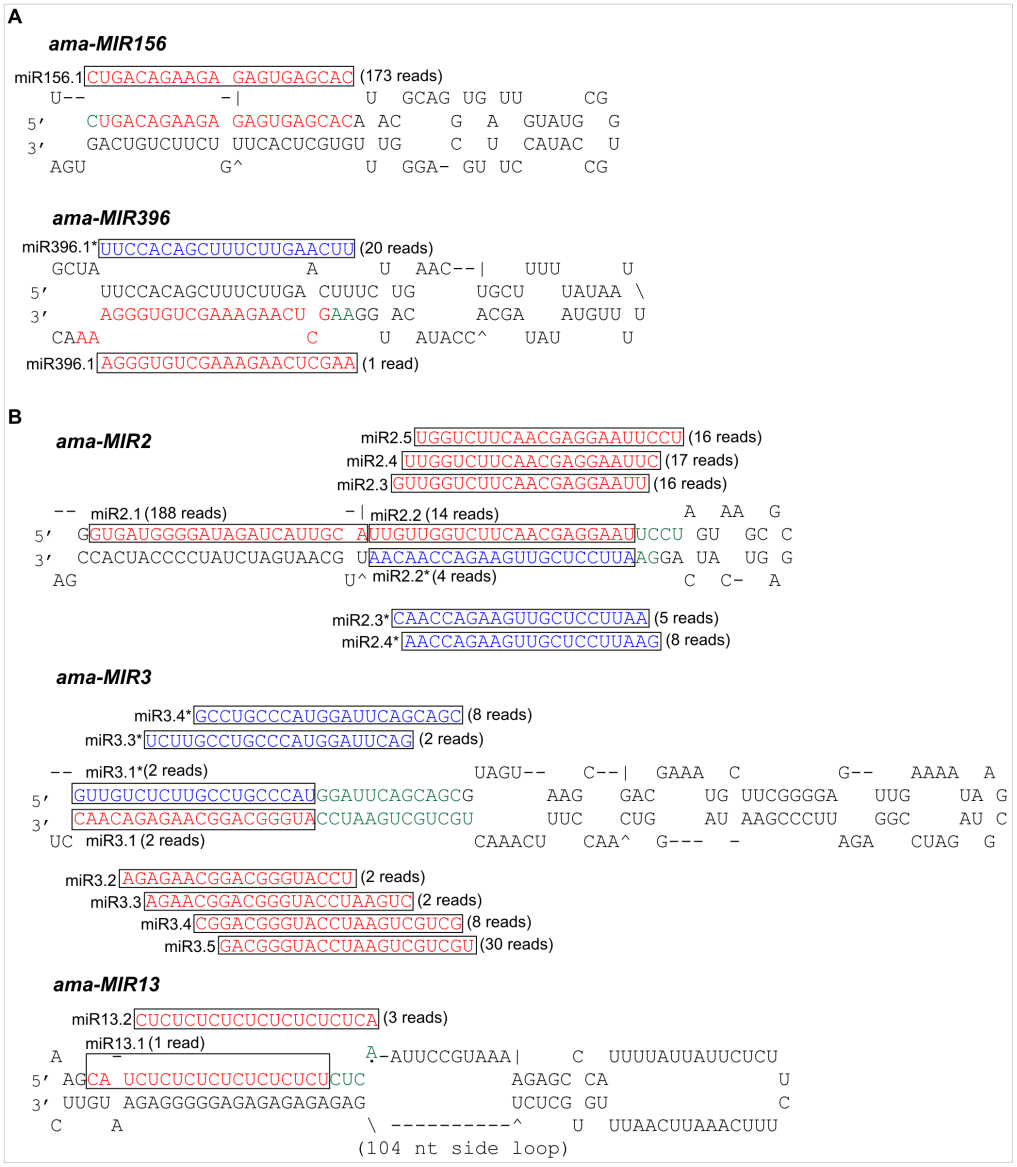
Mangrove miRNA precursors generate multiple miRNAs/miRNA*s. (A) newly produced miRNAs/miRNA*s from conserved *ama-MIR156* and *ama-MIR396* precursors (B) multiple miRNAs/miRNA*s from novel miRNA precursors; *ama-MIR2, ama-MIR3*, *and ama-MIR13.* The sequences of the mature miRNAs in precursors are colored red, miRNA*s are colored blue and the nucleotides with different miRNAs/miRNA*s in their precursors are colored green. All newly identified miRNAs/miRNA*s are shown in black boxes and the number of reads is indicated in brackets.

Different miRNAs produced from a single precursor in a phased pattern were recently reported [69]. Here, we report two conserved miRNA precursors, *ama-MIR156* and *ama-MIR396*, which can produce two different miRNAs (miR156.1 and miR396.1) in addition to miR396.1* (Figure 2A), and three novel miRNA precursors, *ama-MIR2, ama-MIR3*, and *ama-MIR13* in *Avicennia marina* that can yield multiple distinct miRNAs/miRNA*s (Figure 2B). *ama-MIR2* produce five different miRNAs (miR2.1, miR2.2, miR2.3, miR2.4 and miR2.5) and 3 miRNA*s (miR2.2*, miR2.3* and miR2.4*). *ama-MIR3* produces 5 different miRNAs (miR3.1, miR3.2, miR3.3, miR3.4 and miR3.5) and 3 miRNA*s (miR3.1*, miR3.3* and miR3.4*). *ama-MIR13* produces two different miRNAs (miR13.1 and miR13.2). We demonstrate that multiple miRNAs could derive from miRNA precursors by sequential processing of Dicer or Dicer-like proteins, which broadly exist in plants (*Oryza sativa*, *Physcomitrella patens*, *Medicago truncatula* and *Populus trichocarpa*) and animals (*Homo sapiens*, *Mus musculus*, *Caenorhabditis elegans* and *Drosophila melanogaster*) [69]. Finally, BLASTN analysis against all nucleotide sequences in the NCBI databases revealed no homologs for miR1, miR5, miR7 and miR8 in other plant species, suggesting that these newly identified putative miRNAs are specific to mangrove species.

The predicted novel miRNAs exhibited much lower expression levels, consistent with the notion that non-conserved miRNAs are often expressed at a lower level than are conserved miRNAs (Table 3). Only one member was identified in each novel miRNA family and four out of 26 novel miRNA families had more than 100 reads. The miR2.1 (188 reads), miR8 (119 reads), miR12 (272 reads) and miR156.1 (173 reads) were the most frequent novel miRNAs in the library. The low abundance of novel miRNAs might suggest a specific role for these miRNAs under various growth conditions, in specific tissues, or during developmental stages. Whether these low-abundant miRNAs are expressed at higher levels in other tissues and organs or whether they are regulated by abiotic stress remains to be investigated.

### Prediction of miRNA Targets in Mangrove

To clarify the biological functions of the newly identified as well as the conserved *Avicennia marina* miRNAs, we searched for putative target genes using the *psRNATarget* program with default parameters [61]. The target genes were found for conserved and novel mangrove miRNA families (Table S3; Table S4). The putative target genes appeared to be involved in a broad range of biological processes. Most of these genes were classified as transcription factors and functional proteins in plant metabolism and environmental stress responses. The predicted targets include homologs of known targets for conserved miRNAs and novel targets. As expected, many conserved miRNAs targeted transcription factors similar to those predicted in *A. thaliana* and other plant species [28], such as those encoding the squamosa promoter binding protein (SBP, miR156), MYBs/TCPs (miR159/319), the auxin response factor (ARF, miR160 and miR167), the NAC domain protein (miR164), HD-ZIPs (miR165/166), nuclear transcription factor Y (miR169), GRAS family (miR170/171) and APETALA2 (AP2, miR172), supporting the idea that conserved plant miRNAs are involved in essential biological processes. Our analysis also predicted miRNAs targeting siRNAs. For example, *Bruguiera gymnorhiza* gi_53821871 and *R. mangle* E5XRSP401AJ46V were annotated based on the high similarity to TAS3 with *Arabidopsis* and *Physalis longifolia* trans-acting siRNA. TAS3 is thought to regulate the expression of auxin (ARF2, ARF4) and ethylene (ETT) response factor genes. In *Arabidopsis*, TAS3 (At3g17185) is, in turn, regulated by miR390 [70]. Other predicted targets included proteins such as ARGONAUTE 1/2 (miR168/403), transport inhibitor response 1 and auxin signaling F-box protein for miR393, APS1 (ATP sulfurylase 3) for miR395, Beta-6 tubulin and laccase for miR397, COPPER/ZINC SUPEROXIDE DISMUTASE 1 for miR398, phosphoric monoester hydrolase for miR399, Plantacyanin and Zinc finger (B-box type) for miR408, and transcripts that coded for unknown proteins. These observations suggested that the function of some well-conserved miRNAs drifted during long periods of plant evolution. Although miRNAs are well conserved over long evolutionary time scales, some of their sequences have changed and display variations in a few nucleotide positions, which provides the chance for some miRNAs to base pair with other target mRNAs, exhibiting species-specific regulatory patterns.

Unlike conserved miRNAs, the targets of novel mangrove miRNAs were not enriched in transcription factors. Their target genes included those encoding the pentatricopeptide repeat-containing protein (PPR), SALT OVERLY SENSITIVE 1 (SOS1), a zinc finger (C3HC4-type RING finger) family protein, heat shock protein, Kinase-associated protein phosphatase, glycosyltransferase, diacylglycerol kinase family protein, disease resistance protein, tubulin beta-5 chain and complex 1 family protein, implying that the corresponding novel miRNAs participate in some specific developmental processes in *Avicennia marina*. We predicted many genes with unknown function and hypothetical genes for miRNA targeting and careful analysis of these potential targets will contribute to our understanding of the role of miRNAs in mangrove species.

### Validation and Expression Patterns of miRNAs and their Target Genes Identified in Mangrove

**Figure 3:**
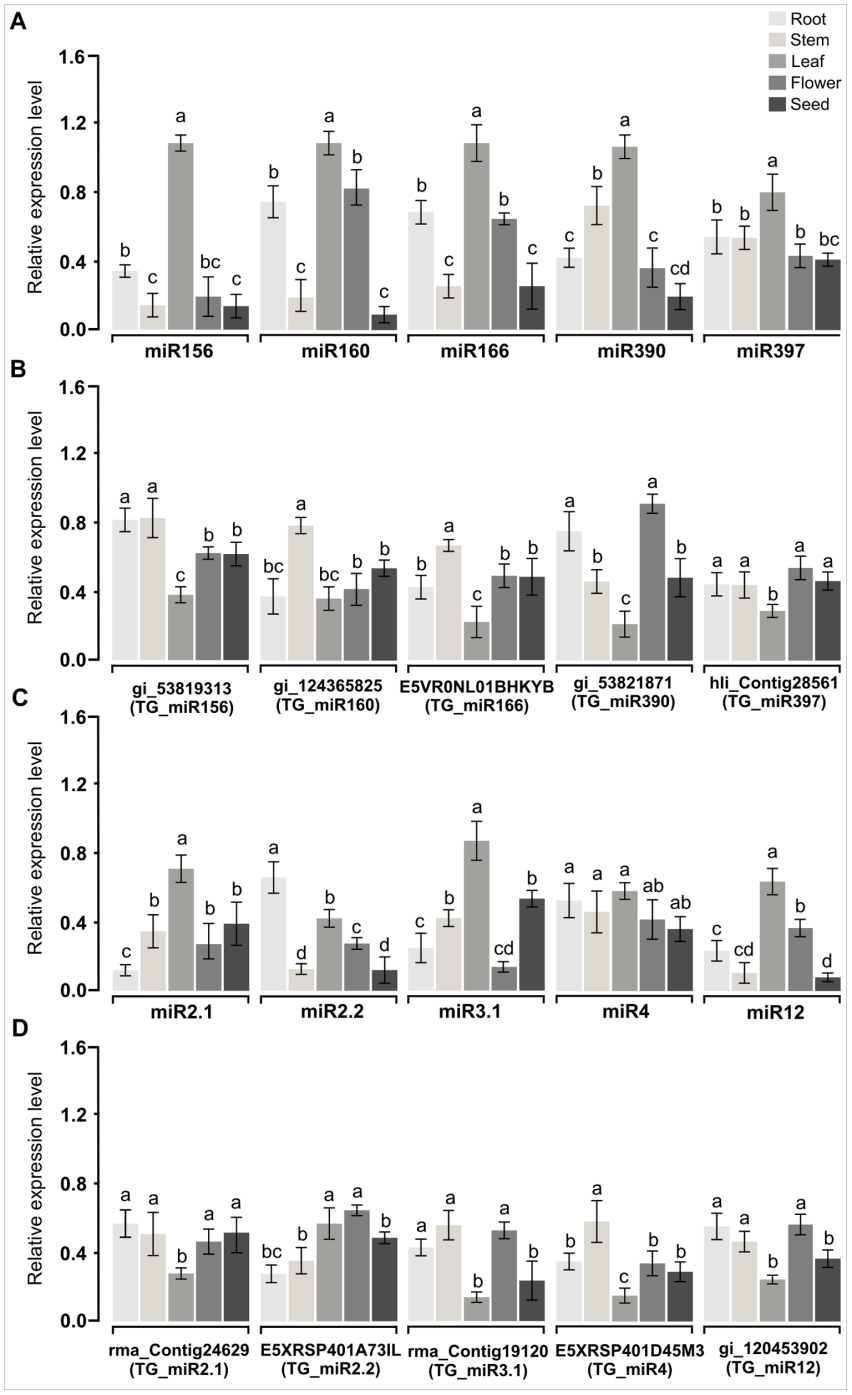
Expression analysis of representative mangrove miRNAs and their target genes. (A) Quantitative stem-loop RT-PCR validation and expression analysis of conserved mangrove miRNAs (B) Quantitative real time PCR expression analysis of conserved mangrove miRNA target genes (C) Quantitative stem-loop RT-PCR validation and expression analysis of novel mangrove miRNAs (D) Quantitative real time PCR expression analysis of novel mangrove miRNA target genes. Error bars indicate the standard deviation of three different technical repeats of each of the two biological replicates. Significant differences at P<0.01 (One Way ANOVA and Duncan test) between samples are indicated with different letters. hli: *Heritiera littoralis*, rma: *Rhizophora mangle*, TG: target gene.

Because sequencing abundance does not necessarily correlate with *in vivo* abundance, we quantified the expression patterns of both the miRNA and their target transcripts, 10 miRNA-target gene pairs were arbitrarily selected for further analyses using qRT-PCR. Different tissues, including roots, stems, leaves, flowers and seeds were used for validation and expression measurement of conserved miRNAs (miR156, miR160, miR166, miR390 and miR397) and novel miRNAs (miR2.1, miR2.2, miR3.1, miR4 and miR12) using stem-loop qRT-PCR as representatives (Figure 3). Stem-loop qRT-PCR is a reliable method for detecting and measuring the expression levels of miRNAs. The stem-loop primers increase the sensitivity of the reactions such that this method can significantly distinguish between two miRNAs with only one single nucleotide change [62].

Based on the threshold cycle (Ct), all conserved miRNAs (miR156, miR160, miR166, miR390 and miR397) and novel miRNAs (miR2.1, miR3.1 and miR12) tested were highly expressed in leaves compared with other tissues (Figure 3A&C). By contrast, miR2.2 was expressed at a higher level in roots, and no significant differences between tissues were found for miR4 (Figure 3C). All miRNAs had much lower expression levels in *Avicennia marina* seeds except for miR 2.1 and miR3.1, which was expressed at a higher level in seeds than in flowers and roots (Figure 3C). These observations suggested that most miRNAs in *Avicennia marina* are expressed preferentially in particular tissues, and most of them are also expressed in multiple tissues.

Expression level of miRNA target genes showed a reliable expression difference between the samples. Compared to miRNA expression levels, expression of their target genes were downregulated in the tissues were they have higher levels of the cognate miRNA and upregulated in the tissues were they have lower expression levels of the cognate miRNA (Figure 3B&D). High accumulation of miRNAs in leaves compared with other tissues showed an efficient downregulation of the cognate target mRNAs, also the low expression level of most miRNAs in *Avicennia marina* seeds allows expression of their mRNA in this type of tissue (Figure 3). Congruent with that the miRNA-transcript target quantifications detected the expected correlations between the miRNA and the target gene expression levels. Our results demonstrated that most of the tested miRNAs were expressed with tissue-specific characteristics and that their preferential expression can provide important clues about where these miRNAs function. The expression analysis of miR156 revealed a tissue-specific expression pattern similar to that found in *Arabidopsis*. In *Avicennia marina,* miR156 had higher expression levels in leaves and roots and lower levels in stems, flowers and seeds. In *Arabidopsis*, miR156 was strongly expressed during seedling development and showed weak expression in mature tissues [71]. MiR156 in *O. sativa* had an expression profile similar to that found in *Arabidopsis* and peanut [13], [71]. Recent results indicate that overexpression of miR156 affects the phase transition from vegetative growth to reproductive growth by causing a rapid initiation of rosette leaves, a severe decrease in apical dominance, and a moderate delay in flowering [72]. MiR160 and miR166 were expressed predominantly in leaves followed by roots and flowers and weakly in seeds and stems in *Avicennia marina*. These expression patterns were closely related to tissue function. The accumulation of miR166 and miR160 could modulate morphological and hormone homeostasis by regulating transcripts of *HD-ZIPIII* and *ARF10.* It has been shown that the miR166/165 group and its target genes regulate diverse aspects of plant development, including apical and lateral meristem formation of shoots, leaf polarity, floral and root development, and vascular development [73]. Moreover, miR160-resistant ARF plants were found to have dramatic developmental abnormalities, and ARF10 was found to regulate floral organ identity and root cap formation was found to be controlled by miRNA-targeted auxin response factors in *Arabidopsis* [74]. MiR390 showed higher expression levels in leaves and stems, and lower levels in roots, flowers and seeds. It has been shown that miR390 and its target gene TAS3 (ARF) regulate diverse aspects of plant development, including leaf polarity [70]. MiR397 was expressed predominantly in leaves and no significant differences in its expression were found between roots, stems, flowers and seeds in *Avicennia marina*. Recent results indicate that miR397 can be induced by environmental factors and not only by developmental processes and it has been shown that miR397 was upregulated by general stress treatments [75].

### MiRNA-guided Target Cleavage in Mangrove

**Figure 4:**
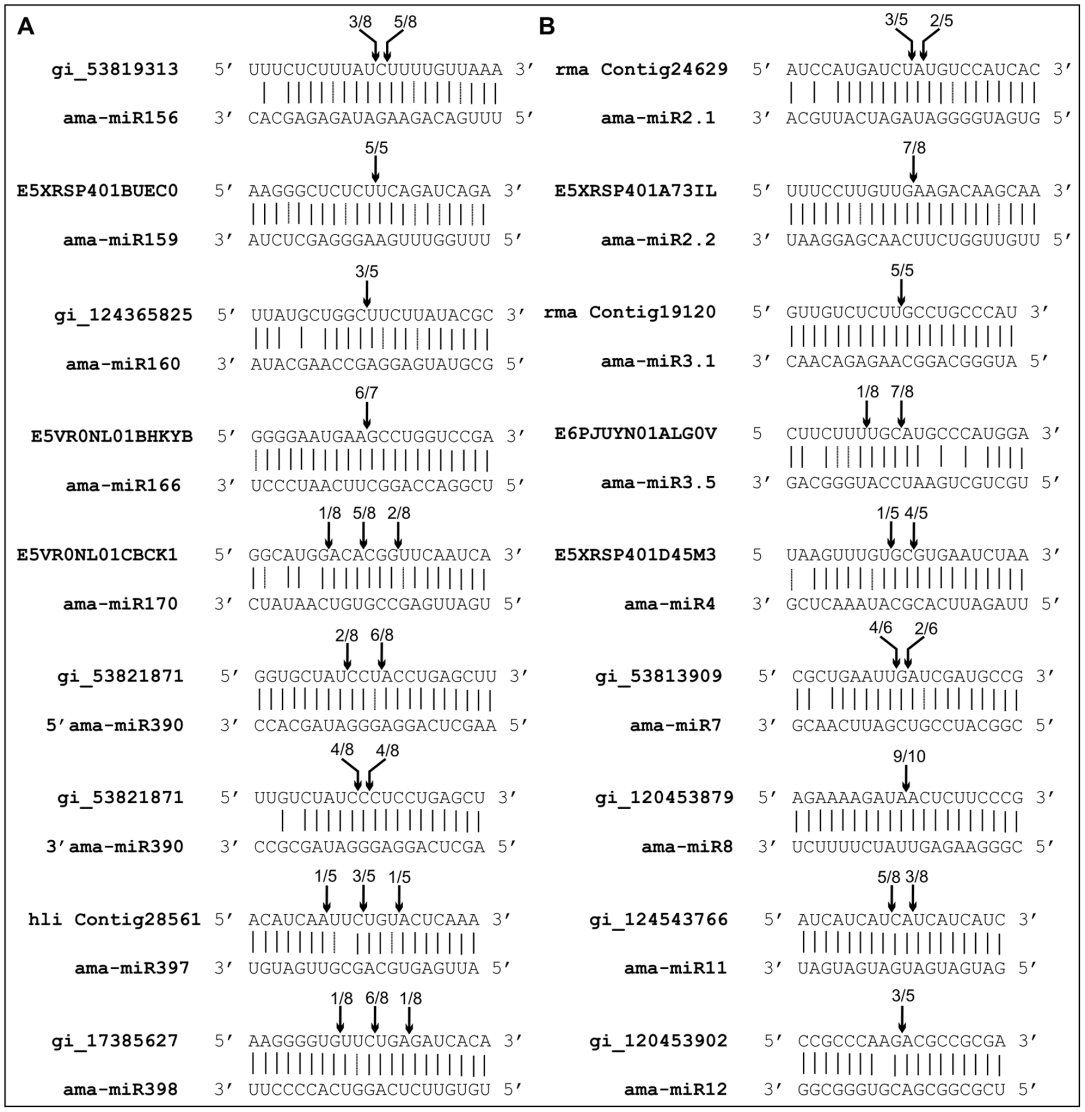
Experimental validation of the predicted miRNA target genes of mangrove *Avicennia marina* . (A) Conserved mangrove miRNAs (B) Novel mangrove miRNAs. MiRNA-guided sites were identified by 5' RACE-PCR. PCR products were cloned and sequenced. Arrows indicate mapped cleavage positions with the frequency amongst clones sequenced. Target gene sequences are shown on top of the miRNA sequences.

miRNAs control the expression of cognate target genes by binding to reverse complementary sequences, resulting in cleavage or translational inhibition of the target RNAs [75]. In this study miRNA target genes were computationally predicted for both conserved and candidate novel miRNAs, the expression of these miRNAs should cause cleavage of the cognate mRNAs within the region complementary to the miRNA sequences. Target sites in plant mRNAs normally share high sequence complementarity to the respective miRNA [2], [27], [28]. Normally, in plants, cleavage within a target transcript that is mediated by a 21-nt miRNA occurs between positions 10 and 11 with respect to the miRNA sequence [2], [27], [28]. To prove this, we performed 5′RACE-PCRs to detect specific mRNA cleavage products. 5′RACE assays were done using pooled total RNA from several mangrove tissues and gene-specific primer sets (Table S1). Cleavage sites of the predicted target genes for both conserved miRNAs (miR156, miR159, miR160, miR166, miR170, miR390, miR397 and miR398) and novel miRNAs (miR2.1, miR2.2, miR3.1, miR3.5, miR4, miR7, miR8, miR11 and miR12) were identified (Figure 4). The PCR products corresponding to the expected size of cleavage products were cloned and sequenced to determine the precise mRNA cleavage sites. Sequencing of 5′ ends revealed cleavage events directed by both conserved and novel miRNAs at a predominant position at the center of the miRNA/mRNA interaction (position 10 to 11) (Figure 4). Also there were several and minority cleavage sites distributed along the *ama-miR170*/E5VR0NL01CBCK1, *5′ama-miR390*/gi_53821871, *ama-miR397*/*H. littoralis* contig28561, *ama-miR398*/gi_17385627, *ama-miR3.5*/E6PJUYN01ALG0V, *ama-miR4*/E5XRSP401D45M3 and *ama-miR11*/gi_124543766 duplexes (Figure 4).

All targets regulated by previously annotated miRNAs and novel miRNAs identified in this study were subjected to GO analysis to identify gene functions [64]. We found that 48 genes were involved in 84 different molecular functions, 44 genes took part in 103 biological processes and 47 genes were part of 47 cellular components (Table S5). The GO biological process suggests that the spectrum of action by miRNAs seems to be extremely wide and includes various aspects of development, adaptive responses to stresses, hormone signaling, cell cycle, circadian rhythm, fatty acid biosynthesis, and carbohydrate metabolism. Most miRNAs do not function independently but rather are involved in overlapping regulatory networks.

**Figure 5:**
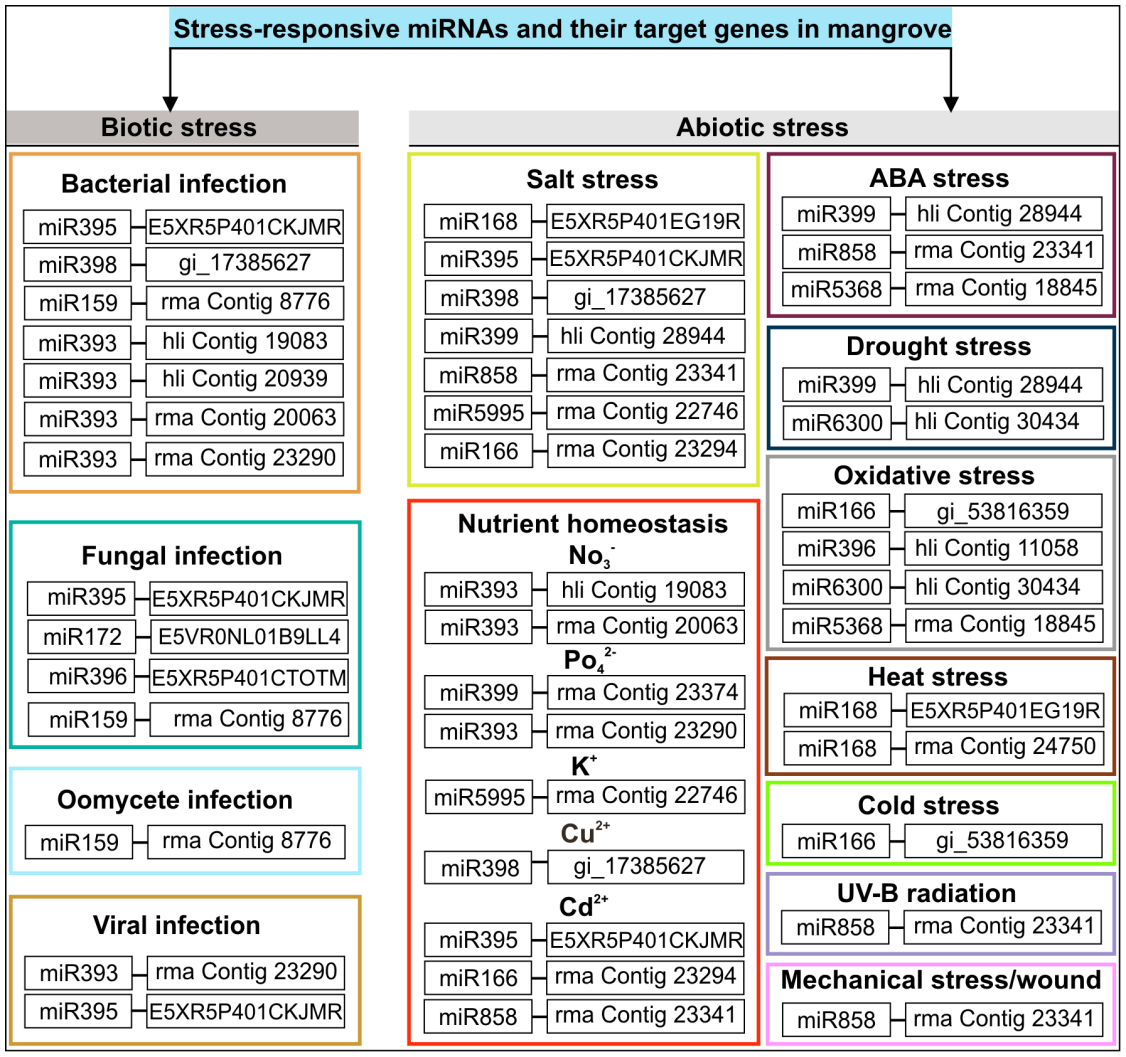
Summary of stress-regulated miRNAs and their target genes from mangrove *Avicennia marina*. miRNAs and their targets are categorized based on the stress that they respond to. BLASTX analysis of the plant protein database (UniProt) was used to identify the nearest homologs and their likely functions were further characterized using the UniProt-GO Annotation database; hli: *Heritiera littoralis*, rma: *Rhizophora mangle*. See Table S5 for the complete list.

One obvious feature of the adaptation to stress is a change in gene expression profiles for genes involved in a broad spectrum of biochemical, cellular, and physiological processes [76]. Under optimal conditions, all resources are used for supporting plant growth and development. Under stress, however, growth and development are stalled, and resources are mobilized toward adaptive responses to stress. Several stress-regulated miRNAs have been identified in model plants under various biotic and abiotic stress conditions, including nutrient deficiency, drought, cold, salinity, bacterial infection, UV-B radiation and mechanical stress [75]. In mangrove species, most conserved miRNAs target mRNAs encoding diverse families of transcription factors. For example miR156, miR159/319, miR160, miR166, and miR169 target SBPs, MYBs/TCPs, ARFs, HD-ZIPs, and the NFY subunit, respectively, but miR168, miR393, miR395, and miR398 target mRNAs encoding AGO1, TIR1, ATS/APS, and CSD1, respectively. The level of these conserved miRNAs appears to be regulated under stress conditions [74]. Our GO analysis demonstrated that 20 miRNA target genes appear to be stress-regulated (Figure 5). The stress-responsive miRNAs and their target genes in mangrove may be involved in many pathways that reprogram complex metabolic and physiological processes, suggesting that plant growth and development are modulated during stress.

### Conclusions

In summary, small RNA profiling using high-throughput sequencing is, in most cases, a straightforward process for model plants for which genomic and biocomputing tools are widely implemented. However, small RNA analysis is challenging for plant species, such as mangrove, for which the genome is largely unknown. In this study, we identified large numbers of miRNAs from mangrove, analyzed their expression levels, and predicted the putative targets of these miRNAs. It will be very important to experimentally characterize these miRNAs and their downstream targets, as this will lead to a better understanding of the functional relationships and mechanisms of miRNAs in the regulatory network, particularly in response to abiotic stresses. Because mangroves have a well-developed ability to adapt to abiotic stresses, this plant is an excellent model system for such an analysis. Additionally, the deep sequencing approach to miRNA discovery suggests that a significant number of novel miRNAs remain to be discovered and characterized. The current study can help to initiate further studies of mangrove miRNA regulatory mechanisms and help to elucidate the important roles of miRNAs in mangrove, including the responses of mangrove to abiotic stresses.

## Supporting Information

Figure S1 **Prediction of secondary structures for some conserved miRNAs in** ***Avicennia marina*** **.** The putative miRNA sequences identified through deep sequencing of small RNAs are highlighted in red and miRNA* sequences are highlighted in blue.
(PDF)

Figure S2 **Prediction of secondary structures of novel miRNA candidates in** ***Avicennia marina*** **.** The putative miRNA sequences identified through deep sequencing of small RNAs are highlighted in red and miRNA* sequences are highlighted in blue.
(PDF)

Table S1 Primers used in this study.
(DOC)

Table S2 Identified conserved miRNAs in *Avicennia marina*.
(DOC)

Table S3 Predicated targets of conserved miRNAs in *Avicennia marina*.
(DOC)

Table S4 Predicated targets of novel miRNA candidates in *Avicennia marina*.
(DOC)

Table S5 **Gene Ontology categories of miRNA target genes in** ***Avicennia marina*** **.** GO terms in the molecular function category are highlighted in blue, GO terms in the biological process category are highlighted in green and GO terms in the cellular location category are highlighted in gray.
(XLS)
