# Supplementary material for: Identification and Analysis of Red Sea Mangrove (Avicennia marina) microRNAs by High-Throughput Sequencing and Their Association with Stress Responses
Source: PLoS One. 2013 Apr 8;8(4):e60774. doi: 10.1371/journal.pone.0060774 (PMC3620391; doi:10.1371/journal.pone.0060774)
Supplement: Figure S1 — Prediction of secondary structures for some conserved miRNAs in Avicennia marina . The putative miRNA sequences identified through deep sequencing of small RNAs are highlighted in red and miRNA* sequences are highlighted in blue. (PDF) [file pone.0060774.s001.pdf]

Figure S1

ama-MIR156

```
  U--           -|          U  GCAG UG UU     CG
5'   CUGACAGAAGA GAGUGAGCACA AC    G  A  GUAUG  G
3'   GACUGUCUUCU UUCACUCGUGU UG    C  U  CAUAC  U
  AGU           G^          U  GGA- GU UC     CG
```

ama-MIR156

```
  GUU         -              U  -|  U     AUUUU
5'   UGACAGAAG AUAGAGAGCACAGA GA UGA GUGCA     U
3'   ACUGUCUUC UAUCUCUCGUGUUU CU ACU UACGU     G
  ACU         G              C  C^  C     CUUAG
```

ama-MIR159

```
  GA            GA       UA   GU   U U--|      AUU           C    GGUC  A           C  CAUCUUCU GG A
5'  GUGGAGCUCCUU  AGUCCAA  GAG  UCU GC   GGGUAG   UAGCUUCUGAG UAUG    CC CAGCCUUAUCC AU        G  G C
3'  CAUCUCGAGGGA  UUAGGUU  UUC  AGA UG   UCCAUU   GUCGAGGACUC AUAC    GG GUUGGGAUAGG UG        C  U U
  A-            AG       UG   UU   U UCG^      AC-           U    GUUC  C           U  -------- UU U
```

ama-MIR171

```
  GG---|       UG         A     A  AUU      UCA
5'     GAUAUUGG  CGGUUCAAU AGAAA CA   GCUCAG   \
3'     CUAUAACC  GCCGAGUUA UUUUU GU   CGGGUU   U
  GUGCA^       GU         G     C  CU-      UUG
```

ama-MIR396

```
  GCUA                 A     U  AAC--|   UUU     U
5'    UUCCACAGCUUUCUUGA CUUUC UG     UGCU   UAUAA \
3'    AGGGUGUCGAAAGAACU GAAGG AC     ACGA   AUGUU U
  CAAA                 C     U  AUACC^   UAU     U
```

ama-MIR396

```
  AU    -|         C          UC   UA  U     UUUG  U    CGG
5'  UCUU CCACAGCUUU UUGAACUGCA  UCU  GA UUGUU    CA GCCA   \
3'  AGAA GGUGUCGAAA AACUUGGCGU  AGG  UU AGCAG    GU CGGU   U
  U-    G^         U          UU   UG  C     ----  -    CUU
```

ama-MIR397

```
  AUC           C            -     .-GUUUAUG| U
5'   AUUGAGUGCAG GUUGAUGAAUUC UUUCA         GC U
3'   UAAUUCACGUC CGACUGCUUAAG AGAGU         CG A
  UAC           -            U     \ -------^ U
                                (21 nt side loop)
```

ama-MIR398

```
  CAA    A     A     U     GU-  U    CCU--|     AAA
5'   CAGG GCGAC UGAGA CACAU   GG CGGU     AGGUUU   A
3'   GUCC CGCUG ACUCU GUGUA   CC GUCA     UCCAAA   C
  GAC    C     G     U     GUU  U    CCUAU^     GUU
```
