# Supplementary material for: Identification and Analysis of Red Sea Mangrove (Avicennia marina) microRNAs by High-Throughput Sequencing and Their Association with Stress Responses
Source: PLoS One. 2013 Apr 8;8(4):e60774. doi: 10.1371/journal.pone.0060774 (PMC3620391; doi:10.1371/journal.pone.0060774)
Supplement: Figure S2 — Prediction of secondary structures of novel miRNA candidates in Avicennia marina . The putative miRNA sequences identified through deep sequencing of small RNAs are highlighted in red and miRNA* sequences are highlighted in blue. (PDF) [file pone.0060774.s002.pdf]

# Figure S2

***ama-MIR1***

```
  GC-|                          CAA
5'   CUUCUAUAGUUUAGGUAACUU    A
3'   GAAGAUAUCAAAUCCAUUGAA    C
  AUA^                          ACA
```

***ama-MIR2***

```
                                  miR2.5 UGGUCUUCAACGAGGAAUUCCU
                                 miR2.4 UUGGUCUUCAACGAGGAAUUC
                                miR2.3 GUUGGUCUUCAACGAGGAAUU
  --  miR2.1                     -| miR2.2                      A   AA   G
5'   GGUGAUGGGGAUAGAUCAUUGC  AUUGUUGGUCUUCAACGAGGAAUUCCU  GU   GC  C
3'   CCACUACCCCUAUCUAGUAACG  UAACAACCAGAAGUUGCUCCUUAAGGA  UA   UG  G
  AG                          U^ miR2.2*                    C   C-   A
                                miR2.3* CAACCAGAAGUUGCUCCUUAA
                                 miR2.4* AACCAGAAGUUGCUCCUUAAG
```

***ama-MIR3***

```
            miR3.4* GCCUGCCCAUGGAUUCAGCAGC
        miR3.3* UCUUGCCUGCCCAUGGAUUCAG
  -- miR3.1*                               UAGU--    C--|   GAAA   C          G--    AAAA   A
5'  GUUGUCUCUUGCCUGCCCAUGGAUUCAGCAGCG       AAG    GAC     UG  UUCGGGGA    UUG     UA  G
3'  CAACAGAGAACGGACGGGUACCUAAGUCGUCGU       UUC    CUG     AU  AAGCCCUU    GGC     AU  C
  UC miR3.1                                CAAACU    CAA^   G---    -          AGA    CUAG   G
   miR3.2 AGAGAACGGACGGGUACCU
     miR3.3 AGAACGGACGGGUACCUAAGUC
         miR3.4 CGGACGGGUACCUAAGUCGUCG
           miR3.5 GACGGGUACCUAAGUCGUCGU
```

***ama-MIR4***

```
  UUA                       UC      CAAUCACCAACCC       AA|   CCAAAAGUUU
5'   AGUUUGUGCGUGAAUCUAAC   AGAUU                 AGAUUC  UCA            \
3'   UCAAAUACGCACUUAGAUUG   UCUAG                 UCUGAG  AGU            U
  UGC                       U-      ACCC---------       AC^   UUCCUGUACC
```

***ama-MIR5***

```
  A    C                     UUU----    C    U-----      CAUAA---------        AUAU-       C----   C-    -|  U
5' GGUG CUUUUUUUUUUUUUUUUU          UAU  UGC       UGAAA                 AGGAAAA      UCCAUU      UG   ACU  GGU  A
3' UUAC GAAAAGAAAGAGAAAAGA          AUA  AUG       ACUUU                 UCCUUUU      AGGUAA      AC   UGA  CCA  U
  U    A                     CACCACU    C    UUUAGU      UUUCCAAUUCCCUG        GUGCU       UUUUU   UU    U^  U
```

***ama-MIR6***

```
  UC|         U   -             U
5'  GGGGUAUUA  GA  CUUUUUAUUC  U
3'  CUCCGUAGU  CU  GAAGGAUAAG  U
  UU^         -   A             U
```

***ama-MIR7***

```
  AAG          -|    C       GA-    UA   AUG
5'   UGUUGAAUC  AUGG  AUGCCG    GGC   CU    \
3'   GCAACUUAG  UGCC  UACGGC    UCG   GA    C
  CGA          C^    -       AAC    UA   GGA
```

***ama-MIR8***

```
  G-|               U      GUUUAACAGC
5’  CGGGAAGAGUUAUC UUUCU           \
3’  GCCCUUCUCAAUAG AAAGG           C
  UA^               U      UCCCACCCGC
```

***ama-MIR9***

```
  UUU           -        GU   A   AUAAGUGGGA--|   G
5’   GUGAAAUGAC UUGAGAG   GU  GG              GCC  A
3’   CAUUUUAUUG AAUUUUC   CA  CC              CGG  A
  AUU           C        AU   -   AUAAAGUGAAAG^   A
```

***ama-MIR10***

```
  C--                  C          CUCUC----       CU           CCGCCGUUCU    G—  -|      A
5’   UCUCUCUCUCUCUCU UCUCUCUCU          UCUCUCU  CUCUCUCUCU           AGUC    GC GAGAGG A
3’   AGAGAGAGAGAGAGA AGAGAGAGA          AGAGAGG  GAGAGAGAGA           UCAG    CG CUCUUC A
  GAA                  U          AAAAAUUAU       UU           C---------    ACUG  A^      G
```

***ama-MIR11***

```
  GUU|                     U GU
5’   GCAUUAUUAUUAUUAUUAUUG U  U
3’   CGUAGUAGUAGUAGUAGUAGU G  G
  ACU^                     U UU
```

***ama-MIR12***

```
  GGA|       A          U
5’   UCGCGGCG CGUGGGCGG U
3’   AGCGCUGC GCGCCCGUC C
  AAG^       A          G
```

***ama-MIR13***

```
     miR13.2 CUCUCUCUCUCUCUCUCUCA
      miR13.1
  A     -                         A-AUUCCGUAAA|    C  UUUUAUUAUUCUCU
5’ AGCA UCUCUCUCUCUCUCUCUCUCUC              AGAGC CA              U
3’ UUGU AGAGGGGGAGAGAGAGAGAG              UCUCG GU              C
  C    A                          \ ----------^    U  UUAACUUAAACUUU
                                 (104 nt side loop)
```

***ama-MIR14***

```
  CUU       GAG            -------    ------------|   A   U
5’   UUGAUUA   UAGGGGUCGCGA       GAGA            GCAG GCG \
3’   AACUGAU   GUUCUCAGCGCU       CUCU            CGUC CGC A
  AAC       ACU            UAUAUUU    GAGCACUGAUAC^   C   C
```

***ama-MIR15***

```
--                                    CCG---  AA        .-G|         UCCGG    U  GUG--     C
5’ GAGGUUUGAAUAUUAAGUAUGAAUCAUAGUUCGA      CG  AUUUAUUU   UUUCUGUAUU      AAUA GA     UGUGA U
3’ CUCCAAACUUAUAAUUCAUACUUAGUAUCAAGCU      GC  UAGGUAAA   AAAGACAUGA      UUAU CU     ACAUU U
CG                                    UAUGAA  AC        \ -^         UAG--    C  AGUUA     G
                                                       (72 nt side loop)
```
