# Supplementary material for: Identification and Analysis of Red Sea Mangrove (Avicennia marina) microRNAs by High-Throughput Sequencing and Their Association with Stress Responses
Source: PLoS One. 2013 Apr 8;8(4):e60774. doi: 10.1371/journal.pone.0060774 (PMC3620391; doi:10.1371/journal.pone.0060774)
Supplement: Table S1 — Primers used in this study. (DOC) [file pone.0060774.s003.doc]

**Table S1**

| **Primer sequence (5’ → 3’)** | **Description of experiment** |
| --- | --- |
| CCACCTGGTGGCTGCTGGGCTGC | 5’ RACE primer to detect the cleavage product of gi_53819313 (miR156 target gene)  Reverse primer for gi_53819313 quantitative real time PCR |
| CCCGGATTTTTGTGAGATTATTG | 5’ RACE primer to detect the cleavage product of E5XRSP401BUEC0 (miR159 target gene) |
| GCCATTAAACTGGTGTGAACCCAAC | 5’ RACE primer to detect the cleavage product of gi_124365825 (miR160 target gene)  Reverse primer for gi_124365825 quantitative real time PCR |
| CTATGTAATTTAATTCTTTGATAC | 5’ RACE primer to detect the cleavage product of E5VR0NL01BHKYB (miR166 target gene)  Reverse primer for E5VR0NL01BHKYB quantitative real time PCR |
| CTAATTAAAATAATGGATCCTGCATCC | 5’ RACE primer to detect the cleavage product of E5VR0NL01CBCK1 (miR170 target gene) |
| GCTTGCATGAACTATGGAAAGGGATCG | 5’ RACE primer to detect the cleavage product of gi_53821871 (miR390 target gene)  Reverse primer for gi_53821871 quantitative real time PCR |
| GTTTCAGCACGATTCACTGTCG | 5’ RACE primer to detect the cleavage product of *H. littoralis* Contig28561 (miR397 target gene)  Reverse primer for *H. littoralis* Contig28561 quantitative real time PCR |
| GCCCTTCCAACAATGGAATTGGGTCCAG | 5’ RACE primer to detect the cleavage product of gi_17385627 (miR398 target gene) |
| GCAGAAATATGAATGGGGCATTTC | 5’ RACE primer to detect the cleavage product of *R. mangle* Contig24629 (miR2.1 target gene)  Reverse primer for *R. mangle* Contig24629 quantitative real time PCR |
| GGACCTATTTCGTATTCACTATC | 5’ RACE primer to detect the cleavage product of E5XRSP401A73IL (miR2.2 target gene)  Reverse primer for E5XRSP401A73IL quantitative real time PCR |
| CCTCTGGTTACTAAGATGTTTCAG | 5’ RACE primer to detect the cleavage product of *R. mangle* Contig19120 (miR3.1 target gene)  Reverse primer for *R. mangle* Contig19120 quantitative real time PCR |
| CAAGCTGCTGGATAAGCTTGTTGAG | 5’ RACE primer to detect the cleavage product of E6PJUYN01ALG0V (miR3.5 target gene) |
| ACGAGTTTATGCGTGAATCTAACAAG | 5’ RACE primer to detect the cleavage product of E5XRSP401D45M3 (miR4 target gene)  Reverse primer for E5XRSP401D45M3 quantitative real time PCR |
| GGCATTGTATGGCTCCACAACTG | 5’ RACE primer to detect the cleavage product of gi_53813909 (miR7 target gene) |
| CGGAGCGGCCGTCGGTGCAGAT | 5’ RACE primer to detect the cleavage product of gi_120453879 (miR8 target gene) |
| CACATACTTGAGTCCGCGGTAAGG | 5’ RACE primer to detect the cleavage product of gi_124543766 (miR11 target gene) |
| GTCGCCTTTCTTGAGGGCGGCG | 5’ RACE primer to detect the cleavage product of gi_120453902 (miR12 target gene)  Reverse primer for gi_120453902 quantitative real time PCR |
| GGTGGUCTATTATAGCTCAAACAAG | Forward primer for gi_53819313 quantitative real time PCR |
| GGTGCTGTTGCTTTACATGGTATTT | Forward primer for gi_124365825 quantitative real time PCR |
| AUUUCCAUGACGACCAAAAUUAAAG | Forward primer for E5VR0NL01BHKYB quantitative real time PCR |
| CAGGTGAAATCGAGGCATAAGTTTC | Forward primer for gi_53821871 quantitative real time PCR |
| GATGTGATACAGGAGCTGCAAGC | Forward primer for gi_17385627 quantitative real time PCR |
| GCGGAACCACCTTTTCCTGAACT | Forward primer for *R. mangle* Contig24629 quantitative real time PCR |
| GGTTTTTCAATTGTTTTCCTTG | Forward primer for E5XRSP401A73IL quantitative real time PCR |
| GGATTCAGCAGCGTAGTAAGCG | Forward primer for *R. mangle* Contig19120 quantitative real time PCR |
| ACUCCGAAGGCUCCGCCAGCAC | Forward primer for E5XRSP401D45M3 quantitative real time PCR |
| GGACUUGCUAGGGUAUCGACG | Forward primer for gi_120453902 quantitative real time PCR |
| GTCGTATCCAGTGCAGGGTCCGAGGTATTCGCACTGGATACGACGTGCTC | Oligonucleotide for miR156-specific cDNA synthesis (RT primer) |
| GTCGTATCCAGTGCAGGGTCCGAGGTATTCGCACTGGATACGACTGGCAT | Oligonucleotide for miR160-specific cDNA synthesis (RT primer) |
| GTCGTATCCAGTGCAGGGTCCGAGGTATTCGCACTGGATACGACGGGGAA | Oligonucleotide for miR166-specific cDNA synthesis (RT primer) |
| GTCGTATCCAGTGCAGGGTCCGAGGTATTCGCACTGGATACGACGGCGCT | Oligonucleotide for miR390-specific cDNA synthesis (RT primer) |
| GTCGTATCCAGTGCAGGGTCCGAGGTATTCGCACTGGATACGACCATCAA | Oligonucleotide for miR397-specific cDNA synthesis (RT primer) |
| GTCGTATCCAGTGCAGGGTCCGAGGTATTCGCACTGGATACGACTGCAAT | Oligonucleotide for miR2.1-specific cDNA synthesis (RT primer) |
| GTCGTATCCAGTGCAGGGTCCGAGGTATTCGCACTGGATACGACATTCCT | Oligonucleotide for miR2.2-specific cDNA synthesis (RT primer) |
| GTCGTATCCAGTGCAGGGTCCGAGGTATTCGCACTGGATACGACTCTTGC | Oligonucleotide for miR3.1-specific cDNA synthesis (RT primer) |
| GTCGTATCCAGTGCAGGGTCCGAGGTATTCGCACTGGATACGACCGAGTT | Oligonucleotide for miR4-specific cDNA synthesis (RT primer) |
| GTCGTATCCAGTGCAGGGTCCGAGGTATTCGCACTGGATACGACCCGCCC | Oligonucleotide for miR12-specific cDNA synthesis (RT primer) |
| GCGGCGGTGACAGAAGAGAGT | Forward primer for miR156 real time stem-loop PCR |
| CCTCCCGTGCCTGGCTCCCTGT | Forward primer for miR160 real time stem-loop PCR |
| GCGGCGGTCGGACCAGGCTTCA | Forward primer for miR166 real time stem-loop PCR |
| GCGGCGGAAGCTCAGGAGGGAT | Forward primer for miR390 real time stem-loop PCR |
| GCGGCGGTCATTGAGTGCAGCG | Forward primer for miR397 real time stem-loop PCR |
| CCTCCCGGTGATGGGGATAGATC | Forward primer for miR2.1 real time stem-loop PCR |
| GCGGCGGTTGTTGGTCTTCAACG | Forward primer for miR2.2 real time stem-loop PCR |
| CCTCCCGCTGAATCCATGGGCAG | Forward primer for miR3.1 real time stem-loop PCR |
| GCGGCGGTTAGATTCACGCATA | Forward primer for miR4 real time stem-loop PCR |
| CCTCCCGGTCGCGGCGACGT | Forward primer for miR12 real time stem-loop PCR |
| GTGCAGGGTCCGAGGT | Universal reverse primer |
| GATAAAATTGGAACGATACAG | U6 forward primer |
| GGACCATTTCTCGATTTGTGCG | U6 reverse primer |
| CCGCCTCTGGTGTGCACCGGTC | 18s rRNA forward primer |
| CCCCCGGAACCCAAGGACTTTG | 18s rRNA reverse primer |
