# Supplementary material for: Identification and Analysis of Red Sea Mangrove (Avicennia marina) microRNAs by High-Throughput Sequencing and Their Association with Stress Responses
Source: PLoS One. 2013 Apr 8;8(4):e60774. doi: 10.1371/journal.pone.0060774 (PMC3620391; doi:10.1371/journal.pone.0060774)
Supplement: Table S2 — Identified conserved miRNAs in Avicennia marina. (DOC) [file pone.0060774.s004.doc]

**Table S2**

| **miRNA**  **family** | **Sequence (5’-3’)** | **Length**  **(nt)** | **Count** | **Conserved in other plants**  **ath ptr bdi gma zm osa vvi** | | | | | | |
| --- | --- | --- | --- | --- | --- | --- | --- | --- | --- | --- |
| miR156/157 | CGACAGAAGAGAGUGAGCAC | 20 | 55 | ++++ | - | - | - | - | - | - |
|  | CUGACAGAAGAUAGAGAGCAC | 21 | 23 | - | - | - | - | - | - | - |
|  | GCUCACUCUCUAUCUGUCACC | 21 | 1 | - | - | - | - | - | - | - |
|  | GCUCACUUCUCUCUCUGUCAGC | 22 | 7 | - | - | - | - | - | ++++ | - |
|  | GCUUACUCUCUCUCUGUCACC | 21 | 2 | - | - | - | - | - | - | - |
|  | UGACAGAAGAAAGAGAGCAC | 20 | 1 | ++++ | - | - | - | - | - | - |
|  | UGACAGAAGAAAGUGAGCAC | 20 | 22 | - | - | - | - | - | - | - |
|  | UGACAGAAGAGAAUGAGCAC | 20 | 3 | - | - | - | - | - | - | - |
|  | UGACAGAAGAGAGAGAGCAC | 20 | 16 | ++++ | - | - | - | - | - | - |
|  | UGACAGAAGAGAGAGAGCAU | 20 | 1 | - | - | - | - | - | - | ++++ |
|  | UGACAGAAGAGAGCGAGCAC | 20 | 21 | - | - | - | - | ++++ | - | - |
|  | UGACAGAAGAGAGGGAGCA | 19 | 2 | - | - | - | - | - | - | - |
|  | UGACAGAAGAGAGGGAGCAC | 20 | 14 | - | ++++ | - | - | - | - | ++++ |
|  | UGACAGAAGAGAGUGAGCAC | 20 | 12,105 | ++++ | ++++ | ++++ | ++++ | ++++ | ++++ | ++++ |
|  | UGACAGAAGAGAGUGAGCACA | 21 | 433 | - | - | - | - | - | - | - |
|  | UGACAGAAGAGAGUGAGCACU | 21 | 123 | - | - | - | ++++ | - | - | - |
|  | UGACAGAAGAGAGUGAGUAC | 20 | 4 | - | - | - | - | - | - | - |
|  | UGACAGAAGAGGGUGAGCAC | 20 | 1 | - | - | - | - | - | - | - |
|  | UGACAGAGGAGAGUGAGCAC | 20 | 3 | - | - | - | - | - | - | ++++ |
|  | UGUCAGAAGAGAGUGAGCAC | 20 | 3 | - | - | - | - | - | - | - |
|  | UUGACAGAAGAAAGAGAGCAC | 21 | 3 | - | - | - | - | - | - | - |
|  | UUGACAGAAGAGAGAGAGCAC | 21 | 554 | - | - | - | - | - | - | - |
|  | UUGACAGAAGAGAGUGAGCAC | 21 | 1,570 | - | - | - | ++++ | - | - | - |
|  | UUGACAGAAGAUAGAGAGC | 19 | 36 | - | - | - | - | - | - | - |
|  | UUGACAGAAGAUAGAGAGCAC | 21 | 4,213 | ++++ | ++++ | - | ++++ | - | - | ++++ |
|  | UUGACAGAAGAUAGAGGGCAC | 21 | 11 | - | - | - | - | - | - | - |
|  | UUUGACAGAAGAUAGAGAGCAC | 22 | 1 | - | - | - | - | - | - | - |
|  | GCUCUCUAUGCUUCUGUCAUC | 21 | 1 | - | - | - | - | - | - | - |
|  | UGACAGAAGAUAGAGAGCAC | 20 | 81 | ++++ | - | - | - | - | - | - |
| miR158 | UCCCAAAUGUAGACAAAGCA | 20 | 2 | ++++ | - | - | - | - | - | - |
| miR159 | CUUGGAUUGAAGGGAGCUCCC | 21 | 6 | - | - | - | - | - | - | - |
|  | CUUGGAUUGAAGGGAGCUCU | 20 | 3 | - | - | ++++ | - | - | - | - |
|  | CUUGGAUUGAAGGGAGCUCUA | 21 | 84 | - | - | - | - | - | ++++ | - |
|  | GAGCUCCUUGAAGUCCAAUU | 20 | 13 | - | - | - | ++++ | - | - | - |
|  | GAGCUCCUUGAAGUCCAAUUG | 21 | 23 | - | - | - | ++++ | - | - | - |
|  | UUGGAUUGAAGAGAGCUCCC | 20 | 1 | - | - | - | - | - | - | - |
|  | UUGGAUUGAAGGGAGCUCCA | 20 | 17 | - | - | - | - | - | - | - |
|  | UUUGGACUGAAGGGAGCUCUA | 21 | 11 | - | - | - | - | - | - | - |
|  | UUUGGAGUGAAGGGAGCUCUG | 21 | 2 | - | - | - | - | ++++ | - | - |
|  | UUUGGAUUGAAGGGAGCUCUA | 21 | 24,911 | ++++ | ++++ | - | ++++ | - | - | ++++ |
|  | UUUGGAUUGAAGGGAGCUCUG | 21 | 3,338 | - | - | - | - | ++++ | ++++ | - |
|  | UUUGGAUUGAAGGGAGCUCUU | 21 | 115 | ++++ | - | - | - | - | - | - |
|  | UUUGGUUUGAAGGGAGCUCUA | 21 | 24 | - | - | - | - | - | - | - |
| miR160 | GCCUGGCUCCCUGUAUGCCAU | 21 | 1 | - | - | - | - | - | - | - |
|  | GCGUAUGAGGAGCCAAGCAUA | 21 | 28 | - | - | - | ++++ | - | - | - |
|  | UGCCUGGCUCCCUGUAUGCC | 20 | 3 | - | - | - | ++++ | - | - | - |
|  | UGCCUGGCUCCCUGUAUGCCA | 21 | 217 | ++++ | ++++ | ++++ | ++++ | ++++ | ++++ | ++++ |
|  | UGCCUGGCUCCCUGUAUGCCG | 21 | 2 | - | - | - | - | ++++ | ++++ | - |
|  | UGCCUGGCUCCUUGUAUGCCA | 21 | 19 | - | - | - | - | - | - | - |
| miR164 | UGGAGAAGCAGGGCACAUGCC | 21 | 10 | - | - | - | - | - | - | - |
|  | UGGAGAAGCAGGGCACGUGC | 20 | 1 | - | - | - | ++++ | - | - | - |
|  | UGGAGAAGCAGGGCACGUGCA | 21 | 174 | ++++ | ++++ | ++++ | ++++ | ++++ | ++++ | ++++ |
| miR165/166 | UCGGACCAGGCUUCAUCCCC | 20 | 43 | - | - | - | - | - | - | - |
|  | UCGGACCAGGCUUCAUCCCCC | 21 | 1,949 | ++++ | - | - | - | - | - | - |
|  | CGGACCAGGCUUCAUUCCCC | 20 | 84 | - | - | - | ++++ | - | - | - |
|  | CUCGGACCAGGCUUCAUUCCC | 21 | 151 | - | - | ++++ | - | - | - | - |
|  | GGAAUGUUGGCUGGCUCGAGG | 21 | 122 | - | - | - | - | ++++ | ++++ | - |
|  | GGAAUGUUGUCUGGCUCGAGG | 21 | 836 | - | - | - | ++++ | ++++ | ++++ | - |
|  | UCCGGACCAGGCUUCAUUCCC | 21 | 1 | - | - | - | - | - | - | - |
|  | UCGAACCAGGCUUCAUUCCCC | 21 | 262 | - | - | - | - | - | ++++ | - |
|  | UCGGACCAGGCUUCAAUCCCU | 21 | 1 | - | - | - | - | ++++ | ++++ | - |
|  | UCGGACCAGGCUUCAUUCC | 19 | 1,115 | - | - | - | - | - | - | ++++ |
|  | UCGGACCAGGCUUCAUUCCC | 20 | 2,568 | - | - | - | ++++ | ++++ | - | - |
|  | UCGGACCAGGCUUCAUUCCCC | 21 | 72,642,6 | ++++ | ++++ | ++++ | ++++ | ++++ | ++++ | ++++ |
|  | UCGGACCAGGCUUCAUUCCCCC | 22 | 77 | - | - | - | - | - | - | - |
|  | UCGGACCAGGCUUCAUUCCCG | 21 | 36 | - | - | - | ++++ | - | - | - |
|  | UCGGACCAGGCUUCAUUCCCU | 21 | 242 | - | - | - | - | - | ++++ | - |
|  | UCGGACCAGGCUUCAUUCCU | 20 | 1,181 | - | - | - | - | - | - | - |
|  | UCGGACCAGGCUUCAUUCCUA | 21 | 14 | - | - | - | - | - | - | - |
|  | UCGGACCAGGCUUCAUUCCUC | 21 | 83,491 | - | - | - | - | ++++ | ++++ | - |
|  | UCGGACCAGGCUUCAUUCCUU | 21 | 87 | - | ++++ | - | - | - | - | - |
|  | UCGGACCAGGCUUCAUUCUC | 20 | 7 | - | - | - | - | - | - | - |
|  | UCGGAUCAGGCUUCAUUCCUC | 21 | 32 | - | - | - | - | - | ++++ | - |
|  | UCUCGGACCAGGCUUCAUUC | 20 | 1,998 | - | - | - | ++++ | - | - | - |
|  | UCUCGGACCAGGCUUCAUUCC | 21 | 83,700,8 | - | - | ++++ | ++++ | - | - | - |
| miR167 | GGUCAUGCUCUGACAGCCUCACU | 23 | 68 | - | - | - | - | - | - | - |
|  | UGAAGCUGCCAGCAUGAUCU | 20 | 29 | - | - | - | - | - | - | - |
|  | UGAAGCUGCCAGCAUGAUCUA | 21 | 218 | ++++ | ++++ | ++++ | ++++ | ++++ | ++++ | ++++ |
|  | UGAAGCUGCCAGCAUGAUCUAA | 22 | 18 | - | - | - | - | - | - | - |
|  | UGAAGCUGCCAGCAUGAUCUC | 21 | 2 | - | - | - | - | - | - | ++++ |
|  | UGAAGCUGCCAGCAUGAUCUG | 21 | 13 | - | ++++ | - | ++++ | ++++ | ++++ | ++++ |
|  | UGAAGCUGCCAGCAUGAUCUGA | 22 | 1 | - | - | ++++ | ++++ | - | - | - |
|  | UGAAGCUGCCAGCAUGAUCUGG | 22 | 1,743 | ++++ | - | - | - | - | - | - |
|  | UGAAGCUGCCAGCAUGAUCUU | 21 | 12 | - | ++++ | - | ++++ | - | - | - |
|  | UGAAGCUGCCAGCAUGAUCUUA | 22 | 1 | - | - | - | - | - | - | - |
| miR168 | CCCGCCUUGCACCAAGUGAA | 20 | 3 | - | - | - | - | ++++ | - | - |
|  | CCCGCCUUGCAUCAACUGAAU | 21 | 1,242 | - | ++++ | - | - | - | - | - |
|  | CCCGCCUUGCAUCAAGUGAA | 20 | 640 | - | - | - | - | ++++ | - | - |
|  | UCGCUUGGUGCAGAUCGGGAC | 21 | 1 | - | - | ++++ | - | ++++ | ++++ | - |
|  | UCGCUUGGUGCAGGUCGAGAA | 21 | 1 | - | - | - | - | - | - | - |
|  | UCGCUUGGUGCAGGUCGGG | 19 | 23 | - | - | - | ++++ | - | - | - |
|  | UCGCUUGGUGCAGGUCGGGA | 20 | 2,686 | - | - | - | - | - | - | - |
|  | UCGCUUGGUGCAGGUCGGGAA | 21 | 12,063 | ++++ | ++++ | - | ++++ | - | - | ++++ |
|  | UCGCUUGGUGCAGGUCGGGAC | 21 | 66 | - | - | - | - | - | - | - |
| miR169 | CAGCCAAGGAUGACUUGCC | 19 | 1 | - | - | - | ++++ | - | - | - |
|  | CAGCCAAGGAUGACUUGCCGG | 21 | 12 | ++++ | ++++ | ++++ | ++++ | ++++ | ++++ | ++++ |
|  | GGCAGGUUGUUCUUGGCUAC | 20 | 1 | - | ++++ | - | - | - | - | - |
|  | UAGCCAAGGAUGACUUGCCU | 20 | 1 | - | - | - | - | - | - | - |
|  | UAGCCAAGGAUGACUUGCCUA | 21 | 1 | - | ++++ | ++++ | - | ++++ | ++++ | ++++ |
| miR170 | UGAUUGAGCCGUGUCAAUAUC | 21 | 20 | ++++ | - | - | - | - | - | - |
| miR171 | UGAUUGAGCCGUGCCAAUAUC | 21 | 30 | - | ++++ | ++++ | ++++ | ++++ | ++++ | ++++ |
|  | UGUUGGCUCGACUCACUCAGA | 21 | 1 | - | - | - | - | - | - | - |
|  | UGUUGGCUCGGCUCACUCAGA | 21 | 78 | - | - | - | - | ++++ | ++++ | - |
|  | UUGAGCCGCGCCAAUAUCAC | 20 | 2 | - | - | - | - | - | - | - |
|  | UUGAGCCGCGCCAAUAUCACU | 21 | 16 | - | - | - | ++++ | - | - | ++++ |
|  | UUGAGCCGUGCCAAUAUCAC | 20 | 7 | - | - | - | - | ++++ | - | - |
|  | UUGAGCCGUGCCAAUAUCACG | 21 | 26 | ++++ | ++++ | - | ++++ | - | - | - |
| miR172 | AGAAUCUUGAUGAUGCUGCA | 20 | 5 | - | - | - | ++++ | ++++ | - | - |
|  | AGAAUCUUGAUGAUGCUGCAG | 21 | 8 | ++++ | - | - | - | - | - | - |
|  | AGAAUCUUGAUGAUGCUGCAU | 21 | 19 | ++++ | ++++ | ++++ | ++++ | - | ++++ | - |
|  | GGAGCAUCAUCAAGAUUCACA | 21 | 1 | - | ++++ | - | - | - | - | - |
|  | GUAGCAUCAUCAAGAUUCAC | 20 | 7 | - | - | - | ++++ | - | - | - |
|  | GUAGCAUCAUCAAGAUUCACA | 21 | 13 | - | - | - | - | - | - | - |
| miR319 | CUUGGACUGAAGGGAGCUCC | 20 | 136 | - | - | - | - | - | - | - |
|  | CUUGGACUGAAGGGAGCUCCC | 21 | 4,257 | - | - | - | - | - | - | - |
|  | CUUGGACUGAAGGGAGCUCCU | 21 | 378 | - | - | - | - | - | - | - |
|  | UUGGACUGAAAGGAGCUCCU | 20 | 1 | - | - | - | ++++ | - | - | - |
|  | UUGGACUGAAGGGAGCUCC | 19 | 369 | - | - | - | - | - | - | - |
|  | UUGGACUGAAGGGAGCUCCC | 20 | 45,404 | - | ++++ | - | ++++ | - | - | - |
|  | UUGGACUGAAGGGAGCUCCCA | 21 | 15 | - | - | - | - | - | - | ++++ |
|  | UUGGACUGAAGGGAGCUCCCU | 21 | 6,919 | ++++ | - | - | ++++ | - | - | ++++ |
|  | UUGGACUGAAGGGAGCUCCU | 20 | 1,527 | - | ++++ | - | - | - | - | - |
|  | UUGGACUGAAGGGAGCUCCUU | 21 | 764 | ++++ | - | - | - | - | - | - |
|  | UUGGACUGAAGGGAGCUCCUUC | 22 | 15 | - | - | - | ++++ | - | - | - |
|  | UUGGACUGAAGGGUGCUCCC | 20 | 38 | - | - | - | - | ++++ | ++++ | - |
|  | UUGGACUGAAGGGUGCUCCCU | 21 | 7 | - | - | ++++ | - | - | - | - |
|  | UUGGGCUGAAGGGAGCUCCC | 20 | 9 | - | ++++ | - | - | - | - | - |
|  | UUUGGACUGAAGGGAGCUCCU | 21 | 1 | - | - | - | - | - | - | ++++ |
| miR390 | AAGCUCAGGAGGGAUAGCACC | 21 | 5,960 | - | - | - | ++++ | - | - | - |
|  | AAGCUCAGGAGGGAUAGCGCC | 21 | 213 | ++++ | ++++ | ++++ | ++++ | ++++ | ++++ | ++++ |
|  | AGCUCAGGAGGGAUAGCGCC | 20 | 12 | - | - | - | ++++ | - | - | - |
|  | CGCUAUCCAUCCUGAGUUUC | 20 | 1 | - | - | - | ++++ | - | - | - |
| miR393 | AUCAUGCUAUCCCUUUGGAUU | 21 | 7 | - | ++++ | - | - | - | - | - |
|  | UCCAAAGGGAUCGCAUUGAUC | 21 | 52 | - | ++++ | ++++ | ++++ | - | ++++ | ++++ |
|  | UCCAAAGGGAUCGCAUUGAUCC | 22 | 12 | ++++ | - | - | ++++ | ++++ | - | - |
|  | UCCAAAGGGAUCGCAUUGAUCU | 22 | 6 | - | - | - | - | ++++ | ++++ | - |
|  | UUCCAAAGGGAUCGCAUUGAUC | 22 | 49 | - | - | - | ++++ | - | - | - |
| miR394 | UUGGCAUUCUGUCCACCUCC | 20 | 177 | ++++ | ++++ | ++++ | ++++ | ++++ | ++++ | ++++ |
| miR395 | CUGAAGUGUUUGGAGGAACUC | 21 | 1 | - | - | - | - | - | - | - |
|  | UGAAGUGUUUGGGGGAACUC | 20 | 1 | - | - | ++++ | - | - | - | - |
| miR396 | GCUCAAGAAAGCUGUGGGAAA | 21 | 17 | - | - | - | - | - | - | - |
|  | GGUCAAGAAAGCUGUGGGAAG | 21 | 1 | - | - | - | - | ++++ | ++++ | - |
|  | GUUCAAGAAAGCUGUGGAAGA | 21 | 1 | - | - | - | - | ++++ | - | - |
|  | GUUCAAGAAAGCUGUGGGAAA | 21 | 69 | - | - | - | - | - | - | - |
|  | GUUCAAUAAAGCUGUGGGAA | 20 | 8 | - | - | - | - | - | ++++ | - |
|  | GUUCAAUAAAGCUGUGGGAAA | 21 | 1 | - | - | - | - | ++++ | - | - |
|  | GUUCAAUAAAGCUGUGGGAAG | 21 | 116 | - | - | - | ++++ | - | - | - |
|  | UCCACAGCUUUCUUGAACUG | 20 | 582 | - | - | - | ++++ | - | - | - |
|  | UCCCACGGCUUUCUUGAACUU | 21 | 1 | - | - | - | - | - | - | - |
|  | UUCAAUAAAGCUGUGGGAAG | 20 | 128 | - | - | - | ++++ | - | - | - |
|  | UUCCACAGCUUUCUUGAACU | 20 | 1,064 | - | - | - | - | - | - | ++++ |
|  | UUCCACAGCUUUCUUGAACUA | 21 | 9 | - | - | - | - | - | - | ++++ |
|  | UUCCACAGCUUUCUUGAACUG | 21 | 15,484 | ++++ | ++++ | ++++ | ++++ | ++++ | ++++ | ++++ |
|  | UUCCACAGCUUUCUUGAACUGU | 22 | 9 | - | - | - | ++++ | - | - | - |
|  | UUCCACAGCUUUCUUGAACUU | 21 | 21,928 | ++++ | ++++ | ++++ | ++++ | ++++ | ++++ | - |
|  | UUCCACGGCUUUCUUGAACUG | 21 | 5 | - | ++++ | - | - | - | - | - |
|  | UUCCACGGCUUUCUUGAACUU | 21 | 17 | - | ++++ | - | - | - | - | - |
| miR397 | AUUGAGUGCAGCGUUGAUGA | 20 | 5 | - | - | - | - | - | - | - |
|  | AUUGAGUGCAGCGUUGAUGU | 20 | 1 | - | - | - | - | - | - | - |
|  | CCAUUGAGUGCAGCGUUGAUG | 21 | 2 | - | ++++ | - | - | - | - | - |
|  | UCAUUGAGUGCAGCGUUGAUG | 21 | 106 | ++++ | ++++ | ++++ | ++++ | - | ++++ | ++++ |
| miR398 | UGUGUUCUCAGGUCACCCCUG | 21 | 1 | ++++ | - | - | - | - | - | - |
|  | UGUGUUCUCAGGUCACCCCUU | 21 | 7 | ++++ | ++++ | - | ++++ | - | ++++ | ++++ |
|  | UGUGUUCUCAGGUCGCCCCUG | 21 | 11 | - | ++++ | ++++ | ++++ | - | ++++ | ++++ |
| miR399 | CGCCAAAGGAGAGUUGCCCUG | 21 | 1 | - | - | - | - | - | - | ++++ |
|  | UGCCAAAGGAGAAUUGCCC | 19 | 10 | - | - | - | - | - | - | - |
|  | UGCCAAAGGAGAAUUGCCCUG | 21 | 44 | - | ++++ | ++++ | - | ++++ | ++++ | ++++ |
|  | UGCCAAAGGAGAGUUGCCCUA | 21 | 2 | - | ++++ | - | - | - | ++++ | - |
|  | UGCCAAAGGAGAGUUGCCCUG | 21 | 732 | ++++ | - | - | ++++ | ++++ | ++++ | ++++ |
|  | UGCCAAAGGAGAGUUGCCCUU | 21 | 1 | - | - | - | - | - | - | - |
| miR403 | UUAGAUUCACGCACAAACUCG | 21 | 1,525 | ++++ | ++++ | - | - | - | - | ++++ |
|  | UUAGAUUCACGCACAAACUUG | 21 | 1 | - | - | - | ++++ | - | - | - |
| miR408 | AUGCACUGCCUCUUCCCUGGC | 21 | 80 | ++++ | ++++ | - | ++++ | - | - | ++++ |
|  | CUGCACUGCCUCUUCCCUGGC | 21 | 1 | - | - | - | - | ++++ | ++++ | - |
|  | UGCACUGCCUCUUCCCUGGC | 20 | 143 | - | - | - | ++++ | - | - | - |
|  | UGCACUGCCUCUUCCCUGGCU | 21 | 779 | - | - | - | - | - | - | - |
|  | UGCACUGCCUCUUCCCUGGCUG | 22 | 2 | - | - | - | - | - | - | - |
| miR482 | CCUACUCCUCCCAUUCC | 17 | 2 | - | ++++ | - | - | - | - | - |
| miR530 | UGCAUUUGCACCUGCACCUC | 20 | 23 | - | - | - | - | - | - | - |
|  | UGCAUUUGCACCUGCACCUU | 20 | 9 | - | ++++ | - | - | - | - | - |
| miR828 | UCUUGCUCAAAUGAGUAUUCCA | 22 | 1 | - | ++++ | - | ++++ | - | - | ++++ |
| miR858 | UUCGUUGUCUGUUCGACCUUG | 21 | 2,488 | ++++ | - | - | - | - | - | - |
| miR894 | CGUUUCACGUCGGGUUCACC | 20 | 7 | - | - | - | - | - | - | - |
| miR1310 | AGGCAUCGGGGGCGCAACGCCC | 22 | 2 | - | - | - | - | - | - | - |
| miR2111 | UAAUCUGCAUCCUGAGGUCUA | 21 | 1 | - | - | - | - | - | - | ++++ |
|  | UAAUCUGCAUCCUGAGGUUUA | 21 | 7 | ++++ | - | - | ++++ | - | - | - |
| miR2911 | GGCCGGGGGACGGACUGGGA | 20 | 58 | - | - | - | - | - | - | - |
|  | GGCCGGGGGACGGGCUGGGA | 20 | 116 | - | - | - | - | - | - | - |
| miR4995 | AGGCAGUGGCUUGGUUAAGGG | 21 | 2 | - | - | - | ++++ | - | - | - |
| miR5139 | AAACCUGGCUCUGAUACCA | 19 | 45 | - | - | - | - | - | - | - |
| miR5368 | GGACAGUCUCAGGUAGACA | 19 | 36 | - | - | - | ++++ | - | - | - |
| miR6173 | AGCCGUAAACGAUGGAUACU | 20 | 4 | - | - | - | - | - | - | - |
| miR6300 | GUCGUUGUAGUAUAGUGG | 18 | 100 | - | - | - | ++++ | - | - | - |

The abbreviations represent: ath, *Arabidopsis thaliana*; ptr, *Populus trichocarpa*; bdi, *Brachypodium distachyon*; gma, *Glycine max;* zma, *Zea mays*; osa, *Oryza satva*; vvi, *Vitis vinifera*. ++++, miRNA sequences of mangrove were exactly identical to those in other species; -, miRNA sequences of mangrove were not presented in those of other species listed in the table but Known from other plant species.
