# Supplementary material for: Identification and Analysis of Red Sea Mangrove (Avicennia marina) microRNAs by High-Throughput Sequencing and Their Association with Stress Responses
Source: PLoS One. 2013 Apr 8;8(4):e60774. doi: 10.1371/journal.pone.0060774 (PMC3620391; doi:10.1371/journal.pone.0060774)
Supplement: Table S3 — Predicated targets of conserved miRNAs in Avicennia marina. (DOC) [file pone.0060774.s005.doc]

**Table S3**

| **miRNA family** | **Target ID** | **Score** | **MTDB/TAIR Annotation** |
| --- | --- | --- | --- |
| miR156/157 | E5QILSR03BZB5C | 2.5 | SQUAMOSA PROMOTER BINDING PROTEIN-LIKE 11 (SPL11) (AT1G27360.1) |
|  | E5XRSP401D4BHH | 3 | SPL9 (AT2G42200.1) |
|  | E5XRSP401DFHAV | 3 | Ribosomal protein S1 (AT5G30510.1) |
|  | E6PJTYN04EC8IX | 2.5 | Protein of unknown function (DUF581) (AT3G63210.1) |
|  | E5VR0NL01BWOYF | 1 | SPL10 (AT1G27370.1) |
|  | E6PJTYN03DCHB4 | 1 | *Arabidopsis thaliana* isolate CS6626 miR157b primary transcript (miR157b) gene |
|  | gi_53815315 | 1.5 | SPL11 (AT1G27360.1) |
|  | gi_53819313 | 2.5 | SPL11 (AT1G27360.1) |
|  | *H. littoralis* Contig10522 | 1.5 | SPL11 (AT1G27360.1) |
|  | *H. littoralis* Contig8646 | 3 | SPL11 (AT1G27360.1) |
|  | *R. mangle* Contig10221 | 2.5 | SPL11 (AT1G27360.1) |
|  | *R. mangle* Contig16278 | 2 | Ribosomal protein S1 (AT5G30510.1) |
|  | *R. mangle* Contig18186 | 2 | SPL5 [*Arabidopsis thaliana*] |
|  | *R. mangle* Contig24083 | 2.5 | SPL15 (AT3G57920.1) |
|  | *R. mangle* Contig2331 | 3 | Protein of unknown function (DUF581) (AT3G63210.1) |
|  | *R. mangle* Contig24038 | 3 | RNA recognition motif (RRM)-containing protein (AT5G25060.1) |
| miR158 | E6PJTYN03DF81V | 2.5 | Pentatricopeptide repeat-containing protein (PPR) (AT1G64100.1) |
|  | E5XRSP401DC7DB | 3 | Transducin/WD40 repeat-like superfamily protein (AT1G49910.1) |
|  | E5VR0NL01DKLCC | 2.5 | Transducin/WD40 repeat-like superfamily protein (AT1G49910.1) |
|  | gi_114053815 | 3 | Pseudogene of pentatricopeptide (PPR) repeat-containing protein (AT1G62860.1) |
|  | *H. littoralis* Contig21241 | 2 | Transducin/WD40 repeat-like superfamily protein (AT1G49910.1) |
|  | *H. littoralis* Contig21722 | 2 | Hydroxyproline-rich glycoprotein family protein [*Arabidopsis thaliana*] |
|  | *H. littoralis* Contig30893 | 2.5 | Phosphate translocator-related [*Arabidopsis thaliana*] |
|  | *H. littoralis* Contig27899 | 3 | MYB DOMAIN PROTEIN 4 (MYB4); transcription factor [*Arabidopsis thaliana*] |
|  | *H. littoralis* Contig9542 | 3 | Unnamed protein product [*Vitis vinifera*] |
|  | *R. mangle* Contig12167 | 2.5 | Transducin/WD40 repeat-like superfamily protein (AT1G49910.1) |
|  | *R. mangle* Contig10224 | 1.5 | No annotation |
|  | *R. mangle* Contig24116 | 2.5 | FT1 (FUCOSYLTRANSFERASE 1); transferring glycosyl groups [*Arabidopsis thaliana*] |
| miR159 | E6PJTYN04EOVID | 2.5 | GH3.3; indole-3-acetic acid amido synthetase [*Arabidopsis thaliana*] |
|  | E5XRSP401BUEC0 | 2.5 | MYB101 (AT2G32460.1) |
|  | E6PJTYN02CB4O1 | 2.5 | MYB101 (AT2G32460.1) |
|  | E5XRSP401C487O | 3 | MYB101 (AT2G32460.1) |
|  | E5XRSP401D8HL7 | 2.5 | SET domain protein 14 (AT3G61740.1) |
|  | E6PJTYN02CBR9E | 3 | MYB101 (AT2G32460.1) |
|  | E6PJTYN02CHJ8V | 3 | MYB101 (AT2G32460.1) |
|  | E5XRSP401BNC1M | 3 | MYB104 (AT2G26950.1) |
|  | E6PJTYN04EOGUX | 3 | MYB104 (AT2G26950.1) |
|  | E5VR0NL01EZ9FP | 3 | Zinc transporter 10 precursor (AT1G31260.1) |
|  | E5VR0NL01AOXAM | 3 | MYB104 (AT2G26950.1) |
|  | gi_124542703 | 3 | MYB101 (AT2G32460.1) |
|  | *H. littoralis* Contig30901 | 2 | Zinc transporter 10 precursor (AT1G31260.1) |
|  | *H. littoralis* Contig29629 | 2 | bZIP transcription factor family protein [*Arabidopsis thaliana*] |
|  | *H. littoralis* Contig30550 | 2.5 | MYB 104 (AT2G26950.1) |
|  | *H. littoralis* Contig25779 | 3 | MYB 101 (AT2G32460.1) |
|  | *H. littoralis* Contig31406 | 3 | MYB 101 (AT2G32460.1) |
|  | *R. mangle* Contig5888 | 2.5 | Unnamed protein product [*Vitis vinifera*] |
|  | *R. mangle* Contig7157 | 3 | MYB101 (AT2G32460.1) |
|  | *R. mangle* Contig19979 | 3 | MYB 104 (AT2G26950.1) |
|  | *R. mangle* Contig5712 | 3 | SU(VAR)3-9 homolog 7 (AT1G17770.1) |
|  | *R. mangle* Contig8776 | 3 | BAK1 (BRI1-ASSOCIATED RECEPTOR KINASE); kinase [*Arabidopsis thaliana*] |
|  | *R. mangle* Contig12978 | 3 | MYB 104 (AT2G26950.1) |
|  | *R. mangle* Contig4094 | 3 | Calcium ion binding [*Arabidopsis thaliana*] |
| miR160 | gi_124365825 | 3 | No annotation |
|  | *H. littoralis* Contig17597 | 2.5 | No annotation |
|  | *H. littoralis* Contig29060 | 3 | No annotation |
|  | *H. littoralis* Contig4277 | 3 | Auxin response factor 17 (AT1G77850.1) |
|  | *R. mangle* Contig24567 | 2.5 | Auxin response factor 17 (AT1G77850.1) |
|  | *R. mangle* Contig22078 | 3 | Auxin response factor 17 (AT1G77850.1) |
|  | *R. mangle* Contig16857 | 2 | Auxin response factor 17 (AT1G77850.1) |
|  | *R. mangle* contig23274 | 2 | Auxin response factor 10 (AT2G28350.1) |
| miR164 | E5XRSP401A3LK9 | 3 | No annotation |
|  | E5XRSP401CDE8D | 3 | SEC14 cytosolic factor, phosphoglyceride transfer protein, putative [*Arabidopsis thaliana*] |
|  | E6PJTYN01A1HDJ | 3 | Unnamed protein product [*Vitis vinifera*] |
|  | E6PJTYN04D5OUY | 3 | Hypothetical protein [*Arabidopsis thaliana*] |
|  | *A. marina* gi17312651 | 2 | ANAC100/ATNAC5; transcription factor [*Arabidopsis thaliana*] |
|  | *H. littoralis* Contig20266 | 2 | ANAC100/ATNAC5; transcription factor [*Arabidopsis thaliana*] |
|  | *H. littoralis* Contig30237 | 2 | NAC domain transcriptional regulator superfamily protein (AT3G12977.1) |
|  | *H. littoralis* Contig17350 | 3 | NAC domain containing protein 1 (AT1G56010.1) |
|  | *R. mangle* Contig10027 | 2.5 | NAC1 (NAC domain containing protein 21/22); transcription factor [*Arabidopsis thaliana*] |
|  | *R. mangle* Contig15616 | 3 | NAC domain containing protein 1 (AT1G56010.1) |
| miR165/166 | E5VR0NL01BHKYB | 0 | Homeobox-leucine zipper family protein (AT1G30490.1) |
|  | E5VR0NL01D5606 | 2.5 | No annotation |
|  | E5VR0NL01EJOXA | 2 | No annotation |
|  | E5VR0NL01ELPQ2 | 3 | Transducin family protein / WD-40 repeat family protein [*Arabidopsis thaliana*] |
|  | E5VR0NL01EQ8BQ | 3 | Monooxygenase, putative (MO3) [*Arabidopsis thaliana*] |
|  | E6PJTYN01A1MG7 | 3 | No annotation |
|  | E6PJTYN01BEO4V | 3 | ATM (ATAXIA-TELANGIECTASIA MUTATED) [*Arabidopsis thaliana*] |
|  | gi_53816359 | 2.5 | RPL23A (RIBOSOMAL PROTEIN L23A); [*Arabidopsis thaliana*] |
|  | *H. littoralis* Contig19018 | 1 | Homeobox-leucine zipper family protein (AT1G30490.1) |
|  | *H. littoralis* Contig23000 | 3 | AP2/B3-like transcriptional factor family protein (AT1G49475.1) |
|  | *H. littoralis* Contig26343 | 2.5 | Homeobox-leucine zipper family protein (AT1G30490.1) |
|  | *H. littoralis* Contig30657 | 3 | Homeobox-leucine zipper family protein (AT1G30490.1) |
|  | *H. littoralis* Contig4609 | 2.5 | Homeobox-leucine zipper family protein (AT1G30490.1) |
|  | *H. littoralis* Contig7574 | 3 | Homeobox-leucine zipper family protein (AT1G30490.1) |
|  | *R. mangle* Contig16703 | 2 | hypothetical protein [*Arabidopsis thaliana*] |
|  | *R. mangle* Contig21159 | 2 | Homeobox-leucine zipper family protein (AT1G30490.1) |
|  | *R. mangle* Contig21207 | 3 | Protein kinase superfamily protein (AT1G13350.1) |
|  | *R. mangle* Contig21802 | 2 | Protein binding [*Arabidopsis thaliana*] |
|  | *R. mangle* Contig23294 | 3 | RGP3 (REVERSIBLY GLYCOSYLATED POLYPEPTIDE 3); [*Arabidopsis thaliana*] |
|  | *R. mangle* Contig9968 | 0 | *Arabidopsis thaliana* isolate CS6626 miR166b primary transcript (miR166b) gene |
| miR167 | *H. littoralis* Contig19151 | 0 | *Populus trichocarpa* clone Pop1-97L17, complete sequence |
|  | *R. mangle* Contig1700 | 1.5 | Cellulase 2 (AT1G02800.1) |
| miR168 | E5XRSP401EG19R | 2.5 | ATJ3 (*Arabidopsis thaliana* DnaJ homologue 3) |
|  | *H. littoralis* Contig22293 | 2.5 | ARGONAUTE 1 (AT1G48410.1) |
|  | *R. mangle* Contig23604 | 2.5 | carbohydrate binding [*Arabidopsis thaliana*] |
|  | *R. mangle* Contig6387 | 3 | No annotation |
|  | *H. littoralis* Contig18382 | 3 | SKOR (stelar K+ outward rectifier); cyclic nucleotide binding / outward rectifier potassium channel [*Arabidopsis thaliana*] |
|  | *H. littoralis* Contig3922 | 3 | ARGONAUTE 1 (AT1G48410.1) |
|  | *R. mangle* Contig10519 | 3 | ARGONAUTE 1 (AT1G48410.1) |
|  | *R. mangle* Contig24750 | 3 | PRR7 (PSEUDO-RESPONSE REGULATOR 7) [*Arabidopsis thaliana*] |
| miR169 | E6PJTYN02CGB55 | 2 | No annotation |
|  | E5VR0NL01CUURH | 2.5 | Petunia x hybrida partial mRNA for YA1 (nf-YA gene) |
|  | gi_146454505 | 2 | Nuclear factor Y, subunit A8 (AT1G17590.1) |
|  | *H. littoralis* Contig13803 | 1 | Nuclear factor Y, subunit A1 (AT5G12840.1) |
|  | *H. littoralis* Contig10712 | 1.5 | Nuclear factor Y, subunit A8 (AT1G17590.1) |
|  | *H. littoralis* Contig23521 | 2.5 | CCAAT-binding transcription factor(CBF-B/NF-YA)family protein [*Arabidopsis thaliana*] |
|  | *H. littoralis* Contig29037 | 2.5 | Unnamed protein product [*Vitis vinifera*] |
|  | *H. littoralis* Contig694 | 3 | Nuclear factor Y, subunit A8 (AT1G17590.1) |
|  | *R. mangle* Contig6279 | 1.5 | MYB family transcription factor [*Arabidopsis thaliana*] |
|  | *R. mangle* Contig13486 | 2.5 | Nuclear factor Y, subunit A1 (AT5G12840.1) |
|  | *R. mangle* Contig13951 | 2.5 | Nuclear factor Y, subunit A1 (AT5G12840.1) |
|  | *R. mangle* Contig12969 | 2.5 | Nuclear factor Y, subunit A8 (AT1G17590.1) |
|  | *R. mangle* Contig22997 | 2.5 | Eukaryotic translation initiation factor 4F, putative/eIF-4F, putative [*Arabidopsis thaliana*] |
|  | *R. mangle* Contig4906 | 2.5 | Nuclear factor Y, subunit A8 (AT1G17590.1) |
|  | *R. mangle* Contig8931 | 2.5 | Nuclear factor Y, subunit A8 (AT1G17590.1) |
|  | *R. mangle* Contig20066 | 3 | Nuclear factor Y, subunit A1 (AT5G12840.1) |
|  | *R. mangle* Contig24433 | 3 | Nuclear factor Y, subunit A8 (AT1G17590.1) |
| miR170 | E6PJTYN03DN7PF | 2.5 | GRAS family transcription factor (AT2G45160.1) |
|  | E5VR0NL01CBCK1 | 3 | GRAS family transcription factor (AT2G45160.1) |
| miR171 | E5XRSP401EMZWG | 3 | GRAS family transcription factor (AT2G45160.1) |
|  | E5XRSP401CECE6 | 2.5 | GRAS family transcription factor (AT2G45160.1) |
|  | *H. littoralis* Contig29702 | 3 | GRAS family transcription factor (AT2G45160.1) |
|  | *R. mangle* Contig23771 | 3 | GRAS family transcription factor (AT2G45160.1) |
|  | *R. mangle* Contig6228 | 3 | Domain of unknown function (DUF2431) (AT1G55790.1) |
|  | *R. mangle* Contig22875 | 2.5 | GRAS family transcription factor (AT2G45160.1) |
|  | *R. mangle* Contig24130 | 3 | GRAS family transcription factor (AT2G45160.1) |
| miR172 | E5VR0NL01B9LL4 | 2 | APG7 (AUTOPHAGY 7) [*Arabidopsis thaliana*] |
|  | E5XRSP401CBY78 | 3 | APETALA 2 (AT4G36920.1) |
|  | gi_53819936 | 3 | APETALA 2 (AT4G36920.1) |
|  | *H. littoralis* Contig27619 | 3 | APETALA 2 (AT4G36920.1) |
|  | *H. littoralis* Contig8051 | 3 | Target of early activation tagged (EAT) 2 (AT5G60120.1) |
|  | *R. mangle* Contig15554 | 0.5 | APETALA 2 (AT4G36920.1) |
|  | *R. mangle* Contig20325 | 1.5 | APETALA 2 (AT4G36920.1) |
|  | *R. mangle* Contig1231 | 3 | APETALA 2 (AT4G36920.1) |
|  | *R. mangle* Contig22562 | 3 | APETALA 2 (AT4G36920.1) |
|  | *R. mangle* Contig23386 | 3 | APETALA 2 (AT4G36920.1) |
|  | *R. mangle* Contig24374 | 3 | APETALA 2 (AT4G36920.1) |
|  | *R. mangle* Contig7360 | 3 | APETALA 2 (AT4G36920.1) |
| miR319 | E5XRSP401C21CA | 1.5 | Disease resistance protein (CC-NBS-LRR class), putative [*Arabidopsis thaliana*] |
|  | E5XRSP401D8HL7 | 1.5 | MYB 104 (AT2G26950.1) |
|  | E5VR0NL01BQ21I | 3 | No annotation |
|  | gi_124541989 | 2 | MYB 104 (AT2G26950.1) |
|  | *H. littoralis* Contig30550 | 1 | MYB 104 (AT2G26950.1) |
|  | *H. littoralis* Contig31119 | 2.5 | MYB 104 (AT2G26950.1) |
|  | *H. littoralis* Contig23294 | 3 | DNA-binding S1FA family protein [*Arabidopsis thaliana*] |
|  | *H. littoralis* Contig26065 | 3 | MYB 33 (AT2G26950.1) |
|  | *R. mangle* Contig18638 | 2.5 | TCP family transcription factor, putative [*Arabidopsis thaliana*] |
|  | *R. mangle* Contig8091 | 2.5 | TCP family transcription factor, putative [*Arabidopsis thaliana*] |
|  | *R. mangle* Contig12978 | 2.5 | MYB 104 (AT2G26950.1) |
|  | *R. mangle* Contig4094 | 3 | Calcium ion binding [*Arabidopsis thaliana*] |
| miR390 | gi_53821871 | 1.5 | TAS3/TASIR-ARF (TRANS-ACTING SIRNA3); (AT3G17185.1) |
|  | E5XRSP401AJ46V | 1.5 | *Physalis longifolia* trans-acting siRNA, TAS3 (gi225904417) |
|  | *H. littoralis contig* 11121 | 2 | TAS3 (trans-acting siRNA 3); (AT5G49615.1) |
|  | *R. mangle* Contig25268 | 3 | TAS3b (trans-acting siRNA 3b); other RNA (AT5G49615.1) |
| miR393 | *H. littoralis* Contig19083 | 2 | AFB3 (AUXIN SIGNALING F-BOX 3) [*Arabidopsis thaliana*] |
|  | *H. littoralis* Contig20939 | 3 | AFB2 (AUXIN SIGNALING F-BOX 2) [*Arabidopsis thaliana*] |
|  | *R. mangle* Contig20063 | 1 | AFB2 (AUXIN SIGNALING F-BOX 2) [*Arabidopsis thaliana*] |
|  | *R. mangle* Contig23290 | 3 | TIR1 (TRANSPORT INHIBITOR RESPONSE 1) [*Arabidopsis thaliana*] |
|  | *R. mangle* Contig4724 | 3 | NIK1 (NSP-INTERACTING KINASE 1); kinase [*Arabidopsis thaliana*] |
| miR394 | E6PJTYN03C0P03 | 2.5 | F-box family protein |
|  | *R. mangle* contig 5783 | 2.5 | F-box family protein |
| miR395 | E5XRSP401CKJMR | 3 | AS1/MYB91 (ASYMMETRIC LEAVES 1, MYB DOMAIN PROTEIN 91);[*Arabidopsis thaliana*] |
|  | E5XRSP401CH6LL | 3 | ATP sulfurylase 1 (AT3G22890.1) |
|  | gi_53821803 | 1 | APS1 (ATP sulfurylase 3) [*Arabidopsis thaliana*] |
|  | *H. littoralis* Contig16931 | 3 | ATP sulfurylase 1 (AT3G22890.1) |
|  | *H. littoralis* Contig27224 | 3 | ARF16 (AUXIN RESPONSE FACTOR 16); transcription factor [*Arabidopsis thaliana*] |
|  | *R. mangle* Contig24802 | 3 | ATP sulfurylase 1 (AT3G22890.1) |
|  | *R. mangle* Contig24344 | 2.5 | Hypothetical protein [*Arabidopsis thaliana*] |
| miR396 | E5XRSP401CTOTM | 2 | XBCP3 (XYLEM BARK CYSTEINE PEPTIDASE 3); [*Arabidopsis thaliana*] |
|  | E5VR0NL01EKEQP | 3 | MYB 76 (AT5G07700.1) |
|  | *H. littoralis* Contig11058 | 3 | Serine/threonine protein kinase, putative [*Arabidopsis thaliana*] |
|  | *H. littoralis* Contig30321 | 3 | PRF5 (PROFILIN5); actin binding / actin monomer binding [*Arabidopsis thaliana*] |
|  | *R. mangle* Contig2252 | 3 | Unknown [*Populus trichocarpa*] |
|  | *R. mangle* Contig8389 | 3 | No annotation |
| miR397 | E5QILSR03BO52O | 3 | Laccase 2 (AT2G29130.1) |
|  | *H. littoralis* Contig28561 | 3 | CKB2 (casein kinase II beta chain 2); protein kinase CK2 regulator [*Arabidopsis thaliana*] |
|  | *R. mangle* Contig16155 | 3 | Beta-6 tubulin (AT5G12250.1) |
| miR398 | E5XRSP401BH6MX | 3 | No annotation |
|  | gi_17385627 | 3 | CSD1 (COPPER/ZINC SUPEROXIDE DISMUTASE 1) [*Arabidopsis thaliana*] |
| miR399 | *H. littoralis* Contig28944 | 1.5 | ALDEHYDE DEHYDROGENASE 4; [*Arabidopsis thaliana*] |
|  | *R. mangle* Contig23374 | 2 | Phosphoric monoester hydrolase [*Arabidopsis thaliana*] |
| miR403 | E5XRSP401D45M3 | 0 | ARGONAUTE 2 (AT1G31280.1) |
|  | *H. littoralis* Contig31226 | 1.5 | ARGONAUTE 2 (AT1G31280.1) |
|  | *R. mangle* Contig22751 | 2.5 | ARGONAUTE 2 (AT1G31280.1) |
| miR408 | *H. littoralis* Contig30420 | 2.5 | Plantacyanin (AT2G02850.1) |
|  | *H. littoralis* Contig30966 | 3 | Zinc finger (B-box type) family protein [*Arabidopsis thaliana*] |
|  | *R. mangle* Contig5826 | 3 | Plantacyanin (AT2G02850.1) |
| miR482 | gi_124544278 | 1 | Disease resistance protein (CC-NBS-LRR class) family (AT1G12220.1) |
|  | E6PJTYN03DQ50Z | 2 | Disease resistance protein (CC-NBS-LRR class) family (AT1G12220.1) |
|  | *H. littoralis* Contig9621 | 2 | NB-ARC domain-containing disease resistance protein (AT4G27190.1) |
|  | *R. mangle* Contig22994 | 2 | NB-ARC domain-containing disease resistance protein (AT4G27190.1) |
| miR530 | E5XRSP401DGYAA | 3 | *Vitis vinifera* contig VV78X045012.8, whole genome shotgun sequence |
|  | *H. littoralis* Contig31197 | 2 | SSI2 (fatty acid biosynthesis 2); acyl-[acyl-carrier-protein] desaturase [Arabidopsis thaliana] |
| miR828 | E5XRSP401C49VB | 1.5 | MYB 82; DNA binding / transcription factor [*Arabidopsis thaliana*] |
|  | *H. littoralis* Contig11819 | 1 | MYB 114; DNA binding / transcription factor [*Arabidopsis thaliana*] |
| miR858 | *H. littoralis* Contig27402 | 3 | MYB 40; DNA binding / transcription factor [*Arabidopsis thaliana*] |
|  | *R. mangle* Contig23341 | 2.5 | MYB 4; transcription factor [*Arabidopsis thaliana*] |
| miR2111 | *R. mangle* Contig2396 | 0.5 | Kelch repeat-containing F-box family protein [*Arabidopsis thaliana*] |
| miR4995 | *R. mangle* Contig22746 | 2.5 | MRP3 (multidrug resistance-associated protein 3) [*Arabidopsis thaliana*] |
|  | *R. mangle* Contig23843 | 3 | Scarecrow-like transcription factor 11 (SCL11) [*Arabidopsis thaliana*] |
| miR5139 | E5VR0NL01C4YTH | 1.5 | No annotation |
|  | *R. mangle* Contig14704 | 3 | ATB2; oxidoreductase [*Arabidopsis thaliana*] |
| miR5368 | *R. mangle* Contig18845 | 2 | Fructose-bisphosphate aldolase, putative [*Arabidopsis thaliana*] |
|  | *R. mangle* Contig19381 | 2 | Cytochrome c oxidase subunit 3 [*Arabidopsis thaliana*] |
| miR6300 | E6PJTYN03DEYQ4 | 2 | AAO4 (ALDEHYDE OXIDASE 4); aldehyde oxidase [*Arabidopsis thaliana*] |
|  | *H. littoralis* Contig30434 | 2.5 | CDSP32 (Chloroplastic Drought-induced Stress Protein of 32 kd); [*Arabidopsis thaliana*] |
