# Supplementary material for: Identification and Analysis of Red Sea Mangrove (Avicennia marina) microRNAs by High-Throughput Sequencing and Their Association with Stress Responses
Source: PLoS One. 2013 Apr 8;8(4):e60774. doi: 10.1371/journal.pone.0060774 (PMC3620391; doi:10.1371/journal.pone.0060774)
Supplement: Table S4 — Predicated targets of novel miRNA candidates in Avicennia marina. (DOC) [file pone.0060774.s006.doc]

**Table S4**

| **miRNA** | **Target ID** | **Score** | **Annotation in MTDB** |
| --- | --- | --- | --- |
| miR1 | *R. mangle* Contig24966 | 3 | Transcription factor S-II (TFIIS) domain-containing protein [*Arabidopsis thaliana*] |
|  | E5VR0NL01CSU57 | 3 | Hypothetical protein [*Arabidopsis thaliana*] |
| miR2.1 | *R. mangle* Contig24629 | 2.5 | Hypothetical protein [*Arabidopsis thaliana*] |
| miR2.2 | gi_114199326 | 0 | Hypothetical protein [*Vitis vinifera*] |
|  | E5VR0NL01EH7NA | 2.5 | Raphidonema pyrenoidifera partial 18S rRNA gene, strain CCAP 470/5 |
|  | E5XRSP401A73IL | 2.5 | Hypothetical protein [*Arabidopsis thaliana*] |
|  | *R. mangle* Contig24155 | 2.5 | Nucleobase:cation symporter [*Arabidopsis thaliana*] |
|  | *H. littoralis* Contig31254 | 3 | SYP51 (SYNTAXIN OF PLANTS 51) [*Arabidopsis thaliana*] |
|  | *H. littoralis* Contig8334 | 3 | No annotation |
|  | *R. mangle* Contig16168 | 3 | DENN (AEX-3) domain-containing protein [*Arabidopsis thaliana*] |
| miR2.3 | gi_114199326 | 0 | Hypothetical protein [*Vitis vinifera*] |
|  | E5VR0NL01EH7NA | 2.5 | Raphidonema pyrenoidifera partial 18S rRNA gene, strain CCAP 470/5 |
|  | *H. littoralis* Contig26814 | 2.5 | Hypothetical protein [*Vitis vinifera*] |
|  | E5VR0NL01D682E | 3 | Unnamed protein product [*Vitis vinifera*] |
|  | E5VR0NL01ELFQ5 | 3 | Glycosyl hydrolase family 17 protein [*Arabidopsis thaliana*] |
|  | *H. littoralis* Contig4054 | 3 | Zinc finger (C3HC4-type RING finger) family protein [*Arabidopsis thaliana*] |
|  | *H. littoralis* Contig5389 | 3 | CYP96A1 (cytochrome P450, family 96, subfamily A, polypeptide 1); [*Arabidopsis thaliana*] |
|  | *R. mangle* Contig22234 | 3 | EMB1241 (EMBRYO DEFECTIVE 1241); [*Arabidopsis thaliana*] |
|  | *R. mangle* Contig23987 | 3 | HD2A (HISTONE DEACETYLASE 2A); [*Arabidopsis thaliana*] |
|  | *R. mangle* Contig8278 | 3 | PHOT1 (phototropin 1); kinase [*Arabidopsis thaliana*] |
| miR2.4 | gi_114199326 | 0 | Hypothetical protein [*Vitis vinifera*] |
|  | *H. littoralis* Contig26814 | 2 | Hypothetical protein [*Vitis vinifera*] |
|  | *H. littoralis* Contig4054 | 2 | Zinc finger (C3HC4-type RING finger) family protein [*Arabidopsis thaliana*] |
|  | E5VR0NL01EH7NA | 2.5 | Raphidonema pyrenoidifera partial 18S rRNA gene, strain CCAP 470/5 |
|  | *R. mangle* Contig23987 | 2.5 | HD2A (HISTONE DEACETYLASE 2A); nucleic acid binding / zinc ion binding [*Arabidopsis thaliana*] |
|  | E5VR0NL01D682E | 3 | Unnamed protein product [*Vitis vinifera*] |
|  | E5VR0NL01ELFQ5 | 3 | Glycosyl hydrolase family 17 protein [*Arabidopsis thaliana*] |
|  | *R. mangle* Contig19219 | 3 | Hypothetical protein [*Arabidopsis thaliana*] |
| miR2.5 | *H. littoralis* Contig26814 | 2 | Hypothetical protein [*Vitis vinifera*] |
|  | E5VR0NL01EH7NA | 2.5 | Raphidonema pyrenoidifera partial 18S rRNA gene, strain CCAP 470/5 |
|  | *H. littoralis* Contig26361 | 2.5 | Unnamed protein product [*Vitis vinifera*] |
|  | *H. littoralis* Contig6593 | 2.5 | Unnamed protein product [*Vitis vinifera*] |
|  | *R. mangle* Contig9834 | 2.5 | calcium-dependent protein kinase, putative / CDPK, putative [*Arabidopsis thaliana*] |
| miR3.1 | *R. mangle* Contig19120 | 0 | Hypothetical protein [*Vitis vinifera*] |
| miR3.2 | *R. mangle* Contig19120 | 0 | Hypothetical protein [*Vitis vinifera*] |
| miR3.3 | *R. mangle* Contig19120 | 0 | Hypothetical protein [*Vitis vinifera*] |
|  | E5XRSP401C0YCS | 3 | CYCA3;4; cyclin-dependent protein kinase regulator [*Arabidopsis thaliana*] |
|  | *H. littoralis* Contig12509 | 3 | Basic helix-loop-helix (bHLH) family protein [*Arabidopsis thaliana*] |
|  | *R. mangle* Contig15257 | 3 | MS ion channel domain-containing protein [*Arabidopsis thaliana*] |
| miR3.4 | *R. mangle* Contig19120 | 0.5 | Hypothetical protein [*Vitis vinifera*] |
|  | *H. littoralis* Contig14559 | 3 | COL2 (CONSTANS-LIKE 2); transcription factor/ zinc ion binding [*Arabidopsis thaliana*] |
|  | *H. littoralis* Contig17633 | 3 | Fructose-bisphosphate aldolase, putative [*Arabidopsis thaliana*] |
|  | *R. mangle* Contig3881 | 3 | Transcription factor [*Arabidopsis thaliana*] |
| miR3.5 | *R. mangle* Contig19120 | 0 | Hypothetical protein [*Vitis vinifera*] |
|  | E6PJTYN01ALG0V | 1.5 | Pentatricopeptide (PPR) repeat-containing protein [*Arabidopsis thaliana*] |
|  | *H. littoralis* Contig26683 | 1.5 | CCAAT-box binding transcription factor subunit B (NF-YB) (HAP3 ) family [*Arabidopsis thaliana*] |
|  | E6PJTYN04EF6WP | 2 | Oxidoreductase family protein [*Arabidopsis thaliana*] |
|  | *R. mangle* Contig13813 | 2 | VAMP713 (vesicle-associated membrane protein 713) [*Arabidopsis thaliana*] |
|  | E5XRSP401E4H2M | 2.5 | No annotation |
|  | E6PJTYN03C8Z08 | 2.5 | No annotation |
|  | *H. littoralis* Contig7355 | 2.5 | DEGP4 (DEGP PROTEASE 4); serine-type peptidase/ trypsin [*Arabidopsis thaliana*] |
|  | *R. mangle* Contig22584 | 2.5 | Pentatricopeptide (PPR) repeat-containing protein [*Arabidopsis thaliana*] |
|  | *H. littoralis* Contig12064 | 3 | SOS1 (SALT OVERLY SENSITIVE 1); [*Arabidopsis thaliana*] |
|  | *H. littoralis* Contig12172 | 3 | Zinc finger (C3HC4-type RING finger) family protein [*Arabidopsis thaliana*] |
|  | *H. littoralis* Contig24629 | 3 | Transmembrane protein, putative [*Arabidopsis thaliana*] |
|  | *H. littoralis* Contig30943 | 3 | Endomembrane protein 70, putative [*Arabidopsis thaliana*] |
|  | *R. mangle* Contig18433 | 3 | ALA1 (AMINOPHOSPHOLIPID ATPASE1); [*Arabidopsis thaliana*] |
|  | *R. mangle* Contig23229 | 3 | Hypothetical protein [*Arabidopsis thaliana*] |
| miR4 | E5XRSP401D45M3 | 1.5 | No annotation |
|  | *H. littoralis* Contig31226 | 2 | No annotation |
|  | *H. littoralis* Contig29773 | 3 | Hypothetical protein [*Arabidopsis thaliana*] |
|  | *R. mangle* Contig22751 | 3 | Nucleic acid binding [*Arabidopsis thaliana*] |
|  | *R. mangle* Contig25359 | 3 | RNA-binding S4 domain-containing protein [*Arabidopsis thaliana*] |
| miR5 | E5XRSP401DOSRL | 1.5 | Hypothetical protein [*Arabidopsis thaliana*] |
|  | E6PJTYN02CIV54 | 1.5 | *Vitis vinifera*, whole genome shotgun sequence, contig VV78X152892.34, clone ENTAV 115 |
|  | *R. mangle* Contig23615 | 1.5 | *S. latifolia* mRNA, clone CCLS 17 |
|  | E5XRSP401CIJ9K | 2 | No annotation |
|  | E5XRSP401D2K6X | 2 | RHA1 [*Arabidopsis thaliana*] |
|  | E5XRSP401EYPNH | 2 | No annotation |
|  | gi_146454741 | 2 | PDAT (phospholipid:diacylglycerol acyltransferase); [*Arabidopsis thaliana*] |
|  | *H. littoralis* Contig1383 | 2 | *Gossypium arboreum* cDNA clone GA__Ed0083C07r, mRNA sequence |
|  | *H. littoralis* Contig18927 | 2 | Unnamed protein product [*Vitis vinifera*] |
|  | *H. littoralis* Contig30971 | 2 | Haloacid dehalogenase-like hydrolase family protein [*Arabidopsis thaliana*] |
|  | *H. littoralis* Contig31278 | 2 | RPL9 (ribosomal protein L9); structural constituent of ribosome [*Arabidopsis thaliana*] |
|  | *R. mangle* Contig11749 | 2 | *Bruguiera gymnorhiza* cDNA clone BG10, mRNA sequence |
|  | *R. mangle* Contig21225 | 2 | No annotation |
|  | *R. mangle* Contig21634 | 2 | LCBK1 (LONG-CHAIN BASE (LCB) KINASE 1); diacylglycerol kinase [*Arabidopsis thaliana*] |
|  | *R. mangle* Contig22873 | 2 | Major latex allergen Hev b 5 |
|  | *R. mangle* Contig23318 | 2 | BETA ATB BETA (serine/threonine protein phosphatase 2A 55 kDa regulatory subunit B beta isofor); [*Arabidopsis thaliana*] |
|  | *R. mangle* Contig23556 | 2 | Transducin family protein / WD-40 repeat family protein [*Arabidopsis thaliana*] |
|  | *R. mangle* Contig24777 | 2 | Hypothetical protein [*Arabidopsis thaliana*] |
|  | *R. mangle* Contig20021 | 2.5 | GSL03 (GLUCAN SYNTHASE-LIKE 3); [*Arabidopsis thaliana*] |
|  | *R. mangle* Contig22408 | 2.5 | 10 kDa chaperonin, putative [*Arabidopsis thaliana*] |
|  | *R. mangle* Contig23835 | 2.5 | CPK32 (CALCIUM-DEPENDENT PROTEIN KINASE 32); [*Arabidopsis thaliana*] |
| miR6 | E5QILSR08ERB6S | 3 | No annotation |
|  | E5XRSP401D0I18 | 3 | *Oryza sativa* Japonica Group chromosome 3 clone OSJNBa0092N01, complete sequence |
|  | E6PJTYN01ASW0Y | 3 | Protein kinase family protein [*Arabidopsis thaliana*] |
|  | *H. littoralis* Contig24799 | 3 | SCPL9; serine carboxypeptidase [*Arabidopsis thaliana*] |
|  | *H. littoralis* Contig721 | 3 | No annotation |
| miR7 | gi_53813909 | 3 | TUB5 (tubulin beta-5 chain) [*Arabidopsis thaliana*] |
| miR8 | gi_120453879 | 0 | *Arabidopsis thaliana* mRNA for Hypothetical protein, complete cds, clone: RAFL22-85-G17 |
|  | *H. littoralis* Contig27952 | 2.5 | Disease resistance protein (CC-NBS-LRR class), putative [*Arabidopsis thaliana*] |
|  | E5VR0NL01BQB79 | 3 | No annotation |
|  | E5VR0NL01BU18E | 3 | lecithin:cholesterol acyltransferase family protein / LACT family protein [*Arabidopsis thaliana*] |
|  | E5XRSP401B7D3V | 3 | BETA ATB BETA (threonine protein phosphatase 2A 55 kDa regulatory subunit B beta isofor); [*Arabidopsis thaliana*] |
|  | *H. littoralis* Contig6390 | 3 | SCPL29 (serine carboxypeptidase-like 29); serine carboxypeptidase [*Arabidopsis thaliana*] |
| miR9 | E6PJTYN04D8RFC | 0 | Hypothetical protein |
|  | *H. littoralis* Contig253 | 2 | GR_Ea09L12.r; GR_Ea Gossypium raimondii cDNA clone |
|  | E5VR0NL01D4A61 | 2.5 | No annotation |
|  | E5XRSP401CN3SD | 2.5 | No annotation |
|  | *H. littoralis* Contig17349 | 2.5 | Homeobox-leucine zipper protein [Zinnia elegans] |
|  | *H. littoralis* Contig27415 | 2.5 | GR_Ea09L12.r; GR_Ea Gossypium raimondii cDNA clone |
|  | E5VR0NL01EBLWG | 3 | PSK2 (PHYTOSULFOKINE 2 PRECURSOR); growth factor [*Arabidopsis thaliana*] |
|  | E5XRSP401C4JZX | 3 | No annotation |
|  | E5XRSP401C7FWX | 3 | AMP-dependent synthetase and ligase family protein [*Arabidopsis thaliana*] |
|  | E5XRSP401CKS6U | 3 | No annotation |
|  | E5XRSP401DJS1X | 3 | No annotation |
|  | E5XRSP401EKTQ7 | 3 | No annotation |
|  | gi_53816878 | 3 | No annotation |
|  | *H. littoralis* Contig15097 | 3 | No annotation |
|  | *H. littoralis* Contig25950 | 3 | REV7 (Reversionless 7); DNA binding [*Arabidopsis thaliana*] |
|  | *R. mangle* Contig18740 | 3 | Unnamed protein product [*Vitis vinifera*] |
|  | *R. mangle* Contig18895 | 3 | *Bruguiera gymnorhiza* cDNA clone Bg05-09_F16 5-, mRNA sequence |
|  | *R. mangle* Contig22278 | 3 | ATBPM2; protein binding [*Arabidopsis thaliana*] |
|  | *R. mangle* Contig7512 | 3 | IBM1 (INCREASE IN BONSAI METHYLATION 1) [*Arabidopsis thaliana*] |
| miR10 | E5XRSP401CKR8W | 0 | *Bruguiera gymnorhiza* cDNA clone Bg04-13_K17 5-, mRNA sequence |
|  | gi_124542371 | 0 | No annotation |
|  | *R. mangle* Contig13185 | 0 | Complex 1 family protein / LVR family protein [*Arabidopsis thaliana*] |
|  | *R. mangle* Contig15678 | 0 | No annotation |
|  | *R. mangle* Contig24502 | 0 | Hypothetical protein [*Arabidopsis thaliana*] |
|  | *R. mangle* Contig8321 | 0 | Ndr family protein [*Arabidopsis thaliana*] |
|  | *R. mangle* Contig22813 | 0.5 | No annotation |
|  | *R. mangle* Contig22926 | 0.5 | TOM20-3 (TRANSLOCASE OF OUTER MEMBRANE 20 KDA SUBUNIT 3); [*Arabidopsis thaliana*] |
|  | *R. mangle* Contig23156 | 0.5 | 40S ribosomal protein S25 (RPS25B) [*Arabidopsis thaliana*] |
|  | *R. mangle* Contig23657 | 0.5 | Prephenate dehydrogenase family protein [*Arabidopsis thaliana*] |
|  | *R. mangle* Contig23662 | 0.5 | Hypothetical protein [*Arabidopsis thaliana*] |
|  | *R. mangle* Contig23883 | 0.5 | VPS20.2 [*Arabidopsis thaliana*] |
| miR11 | gi_124543766 | 0 | VACUOLAR ATPASE SUBUNIT F; [*Arabidopsis thaliana*] |
|  | *H. littoralis* Contig18176 | 0 | Hypothetical protein [*Arabidopsis thaliana*] |
|  | *H. littoralis* Contig30898 | 0 | ARR6 (RESPONSE REGULATOR 6); [*Arabidopsis thaliana*] |
|  | *H. littoralis* Contig30907 | 0 | Inosine-uridine preferring nucleoside hydrolase family protein [*Arabidopsis thaliana*] |
|  | *H. littoralis* Contig31118 | 0 | Hypothetical protein [*Arabidopsis thaliana*] |
|  | *H. littoralis* Contig31702 | 0 | ARR4 (RESPONSE REGULATOR 4); [*Arabidopsis thaliana*] |
|  | *R. mangle* Contig13425 | 0 | No annotation |
|  | *R. mangle* Contig17661 | 0 | Crs768 lambda ZAPST *Ricinus communis* cDNA clone pcrs768, mRNA sequence |
|  | *R. mangle* Contig18184 | 0 | Unknown [Populus trichocarpa] |
|  | *R. mangle* Contig20480 | 0 | IQD11 (IQ-domain 11); calmodulin binding [*Arabidopsis thaliana*] |
|  | *R. mangle* Contig21737 | 0 | Hypothetical protein [*Arabidopsis thaliana*] |
|  | *R. mangle* Contig21787 | 0 | Unnamed protein product [*Vitis vinifera*] |
|  | *R. mangle* Contig22226 | 0 | Hypothetical protein [*Arabidopsis thaliana*] |
|  | *R. mangle* Contig23986 | 0 | Hypothetical protein |
|  | *R. mangle* Contig24496 | 0 | Reticulon family protein [*Arabidopsis thaliana*] |
|  | *R. mangle* Contig25353 | 0 | VACUOLAR ATPASE SUBUNIT F; [*Arabidopsis thaliana*] |
| miR12 | gi_120453902 | 2 | No annotation |
|  | E5XRSP401AK66M | 3 | EDA9 (embryo sac development arrest 9); [*Arabidopsis thaliana*] |
| miR13.1 | E5QILSR07EC6K1 | 0 | No annotation |
|  | E5VR0NL01A6XGE | 0 | No annotation |
|  | E5VR0NL01B8QYU | 0 | No annotation |
|  | E5XRSP401AXCA8 | 0 | No annotation |
|  | E5XRSP401CSRVH | 0 | No annotation |
|  | E5XRSP401DITMG | 0 | No annotation |
|  | E5XRSP401DMQQA | 0 | Hypothetical protein [*Arabidopsis thaliana*] |
|  | E5XRSP401EDTTA | 0 | No annotation |
|  | E5XRSP401ENN6E | 0 | No annotation |
|  | E5XRSP401EYW4T | 0 | No annotation |
|  | gi_53817753 | 0 | No annotation |
|  | *H. littoralis* Contig11141 | 0 | GH_CHX10C09.x GH_CHX Gossypium hirsutum cDNA clone GH_CHX10C09 5-, mRNA sequence |
|  | *H. littoralis* Contig6740 | 0 | No annotation |
|  | *R. mangle* Contig11526 | 0 | Glycosyl hydrolase family 38 protein [*Arabidopsis thaliana*] |
|  | *R. mangle* Contig17216 | 0 | No annotation |
|  | *R. mangle* Contig18494 | 0 | No annotation |
| miR13.2 | E5VR0NL01CRI44 | 0 | No annotation |
|  | E5XRSP401C7KXU | 0 | No annotation |
|  | E5XRSP401CSGM2 | 0 | No annotation |
|  | E5XRSP401CSRVH | 0 | No annotation |
|  | E5XRSP401CT41I | 0 | No annotation |
|  | E5XRSP401D00BR | 0 | No annotation |
|  | E5XRSP401DMQQA | 0 | Hypothetical protein [*Arabidopsis thaliana*] |
|  | gi_53813175 | 0 | ATP synthase epsilon chain, mitochondrial [*Arabidopsis thaliana*] |
|  | *R. mangle* Contig15845 | 0 | ALDH2C4 (REDUCED EPIDERMAL FLUORESCENCE1); [*Arabidopsis thaliana*] |
|  | *R. mangle* Contig19854 | 0 | Hypothetical protein [*Arabidopsis thaliana*] |
| miR14 | *R. mangle* Contig14401 | 0 | Unnamed protein product [*Vitis vinifera*] |
|  | E5XRSP401AYCV5 | 2.5 | Nodulin MtN21 family protein [*Arabidopsis thaliana*] |
|  | *H. littoralis* Contig15548 | 2.5 | No annotation |
|  | E5VR0NL01BV2MJ | 3 | GHMP kinase family protein [*Arabidopsis thaliana*] |
|  | gi_13873335 | 3 | Mitochondrial / lipoamide dehydrogenase 1 (MTLPD1) [*Arabidopsis thaliana*] |
|  | *R. mangle* Contig10668 | 3 | Heat shock protein 83 |
|  | *R. mangle* Contig22966 | 3 | CXXS1 (c-terminal cysteine residue is changed to a serine 1); [*Arabidopsis thaliana*] |
|  | *R. mangle* Contig23295 | 3 | Hypothetical protein [*Arabidopsis thaliana*] |
|  | *R. mangle* Contig4600 | 3 | D111/G-patch domain-containing protein [*Arabidopsis thaliana*] |
| miR15 | *H. littoralis* Contig31489 | 0 | Gossypium hirsutum cultivar coker 310 FR chloroplast, complete genome |
|  | E5XRSP401C0V6Z | 1 | Hypothetical protein [*Arabidopsis thaliana*] |
|  | E5VR0NL01AUKIR | 2 | No annotation |
|  | E5VR0NL01EIDAL | 2 | No annotation |
|  | *H. littoralis* Contig25212 | 2 | Unnamed protein product [*Vitis vinifera*] |
|  | *H. littoralis* Contig30842 | 2 | SEC14 cytosolic factor family protein / phosphoglyceride transfer family protein [*Arabidopsis thaliana*] |
|  | *R. mangle* Contig1587 | 2 | *Vitis vinifera* contig VV78X134674.6, whole genome shotgun sequence |
|  | *R. mangle* Contig22106 | 2 | Hypothetical protein [*Arabidopsis thaliana*] |
|  | E5XRSP401ARY24 | 2.5 | No annotation |
|  | E5XRSP401BTMTG | 2.5 | No annotation |
|  | E5XRSP401CR097 | 2.5 | No annotation |
|  | E6PJTYN02BVK9P | 2.5 | Leucine-rich repeat family protein / protein kinase family protein [*Arabidopsis thaliana*] |
|  | E6PJTYN04EKMBJ | 2.5 | No annotation |
|  | *H. littoralis* Contig12267 | 2.5 | No annotation |
|  | *H. littoralis* Contig21196 | 2.5 | Mitochondrial substrate carrier family protein [*Arabidopsis thaliana*] |
|  | *H. littoralis* Contig22069 | 2.5 | Zinc finger (C3HC4-type RING finger) family protein [*Arabidopsis thaliana*] |
|  | *H. littoralis* Contig26051 | 2.5 | Hypothetical protein [*Arabidopsis thaliana*] |
|  | *H. littoralis* Contig30924 | 2.5 | Hypothetical protein [*Arabidopsis thaliana*] |
|  | *R. mangle* Contig4836 | 2.5 | E2F3 (E2F TRANSCRIPTION FACTOR-3) [*Arabidopsis thaliana*] |
|  | *R. mangle* Contig5040 | 2.5 | *Bruguiera gymnorhiza* cDNA clone Bg04-25_D03 5-, mRNA sequence |
|  | gi_53813797 | 3 | No annotation |
|  | *R. mangle* Contig23456 | 3 | Hypothetical protein [*Arabidopsis thaliana*] |
